# Mapping the CgrA regulon of *Rhodospirillum centenum* reveals a hierarchal network controlling Gram-negative cyst development

**DOI:** 10.1186/s12864-015-2248-z

**Published:** 2015-12-16

**Authors:** Qian Dong, Mingxu Fang, Sugata Roychowdhury, Carl E. Bauer

**Affiliations:** Molecular and Cellular Biochemistry Department, Indiana University, Bloomington, IN 47405 USA; Present address: Owensboro Cancer Research Program, University of Louisville James Graham Brown Cancer Center, Owensboro, KY 42303 USA; Department of Molecular and Cellular Biochemistry, Indiana University, Simon Hall MSB, 212 S. Hawthorne Drive, Bloomington, IN 47405-7003 USA

**Keywords:** Azospirillum clade, Developmental transcriptomics, Encystment, Desiccation resistance, cGMP regulation

## Abstract

**Background:**

Several Gram-negative species undergo development leading to the formation of metabolically dormant desiccation resistant cysts. Recent analysis of cyst development has revealed that ~20 % of the *Rhodospirillum centenum* transcriptome undergo temporal changes in expression as cells transition from vegetative to cyst forms. It has also been established that one trigger for cyst formation is the synthesis of the signaling nucleotide 3‘, 5‘- cyclic guanosine monophosphate (cGMP) that is sensed by a homolog of the catabolite repressor protein called CgrA. CgrA in the presence of cGMP initiate a cascade of gene expression leading to the development of cysts.

**Results:**

In this study, we have used RNA-seq and chromatin immunoprecipitation (ChIP-Seq) techniques to define the CgrA-cGMP regulon. Our results indicate that disruption of CgrA leads to altered expression of 258 genes, 131 of which have been previously reported to be involved in cyst development. ChIP-seq analysis combined with transcriptome data also demonstrates that CgrA directly regulates the expression of numerous sigma factors and transcription factors several of which are known to be involved in cyst cell development.

**Conclusions:**

This analysis reveals the presence of CgrA binding sites upstream of many developmentally regulated genes including many transcription factors and signal transduction components. CgrA thus functions as master controller of the cyst development by initiating a hierarchal cascade of downstream transcription factors that induces temporal expression of encystment genes.

**Electronic supplementary material:**

The online version of this article (doi:10.1186/s12864-015-2248-z) contains supplementary material, which is available to authorized users.

## Background

The formation of metabolically dormant cysts has been reported for a wide range of proteobacteria [1–3]. Cysts synthesized by Gram-negative cells typically contain a thick exopolysaccharide layer that provides a means of surviving environmental stresses such as desiccation and nutrient deprivation [1, 4]. For the past decade, we have studied Gram-negative cyst cell development by undertaking detailed genetic and biochemical studies of encystment by the thermotolerant α-1 proteobacterium *Rhodospirillum centenum* [4–10]. *R. centenum* is a photosynthetic member of the *Azospirillum* clade, members of which exhibit three morphologically distinct cell types: swim cells, swarm cells and cyst cells [1, 4]. Studies have shown that numerous regulatory factors contribute to the control of *R. centenum* encystment, including several histidine kinases [6, 11], a sigma subunit [6] and a novel Che-like signal transduction cascade [7, 9, 12].

One of the more intriguing encystment regulatory components is the recent demonstration that *R. centenum* produces and secretes 3′, 5′- cyclic guanosine monophosphate (cGMP) upon a nutrient downshift and that this nucleotide is used as a signaling molecule to control encystment [8]. cGMP production is dependent on the transcription factor CgrA which is a homolog of *Escherichia coli* CRP that utilizes cGMP instead of cAMP as a coregulator. CgrA controls cGMP production by activating expression of *gcyB* and *gcyC* coding for putative guanylyl cyclase subunits, as well as its own expression upon binding cGMP [8, 10]. cGMP production thus ramps up in a feed forward loop leading to excretion of increasing amounts of cGMP into the culture supernatant as cells enter encystment [8, 10].

There are several deep sequencing approaches that allow analysis of complex of prokaryotic regulatory networks. For example*,* RNA-seq experiments can compare transcriptome differences between strains of a single bacterial species or global differences in transcription patterns that occur in a single strain grown in different growth conditions [13–16]. Chromatin immunoprecipitation (ChIP), using specific antibodies against transcription factors, can also be applied to identify transcription factor binding sites on a global scale [17]. The combination of ChIP and the detection of enriched DNA fragments on a DNA microarray (ChIP-chip) or by high-throughput sequencing (ChIP-seq) enables global identification of binding sites for nucleoid-associated proteins (NAPs) and transcription regulatory proteins [17, 18]. The major advantage of ChIP-seq over ChIP-chip is that ChIP-Seq offers much higher detection and resolution of target binding sites [19].

ChIP-chip and ChIP-seq experiments with well-studied transcription factors such as CRP have revealed the surprising presence of numerous previously undetected novel binding sites (e.g., within genes and upstream of seemingly unrelated genes), including those considered to be noncanonical in nature. This method was first used in *E. coli* to study the genome-wide distribution of the nucleoid-associated proteins Fis and H-NS [20]. There are currently many ChIP-seq studies in prokaryotes that report the binding sites of transcriptional regulators and sigma factors in diverse species such as *E. coli*, *Mycobacterium tuberculosis*, *Pseudomonas aeruginosa* and *Vibrio cholera* [17, 21–25]*.*

Recently, we utilized RNA-seq technology to profile and quantify gene expression changes that occur as *R. centenum* cells develop into cysts [26]. These results show that ~812 genes exhibited significant changes in expression over a 96 h cyst induction period. Notable changes of expression occur in regulatory genes, metabolic genes and genes involved in cell wall and lipid biosynthesis. With knowledge of baseline changes in expression established, we are now addressing the role of individual transcription factors in controlling this developmental process. In this study, we have utilized a combination of ChIP-seq and RNA-seq to map the CgrA regulon in *R. centenum*. We report that the CgrA regulon comprises 258 genes and have identified numerous genes that are directly regulated by CgrA.

## Results

### Identifying members of the CgrA regulon using RNA-Seq transcriptome profiling

**Fig. 1:**
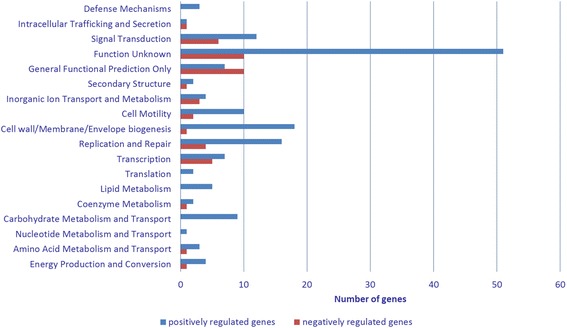
Differentially expressed genes (DEGs) grouped by clusters of orthologous groups (COG). Genes were designated as differentially expressed (DEG) that exhibited a ≥2.5 fold change in expression and a false-discovery-rate adjusted *p*-value of less than 0.05. RNA-seq data set revealed a total of 258 DEGs, among which 199 genes were grouped into 18 unique COG categories. CgrA positively regulated (activated) genes were labeled in blue and CgrA negatively regulated genes (repressed) genes in red

We investigated the extent of the CgrA regulon by comparing genome wide transcription expression profiles of wild type versus *ΔcgrA* strains. For this analysis, we shifted wild type and *ΔcgrA* strains from vegetative CENS growth medium into cyst inducing CENBA medium, extracted cellular RNA and analyzed transcriptome expression levels using RNA-Seq. For this study, we performed detailed analysis of the mRNA expression level, and of CgrA binding to the chromosome, at the 24 h time period post induction as previous analyses have shown that CgrA expression and cGMP production is induced early (within 4 h) in cyst development [10]. We also observed that many early encystment genes are expressed during this time period [26]. RNA-seq data from three biological replicates of wild type and *ΔcgrA* strains contained >53 million (M) strand specific RNA-Seq reads per sample (>70x coverage per nucleotide). Differentially expressed genes (DEGs) were subsequently identified from pair-wise comparisons of sequencing reads between *ΔcgrA* and wild type strains if they exhibited a log_2_fold change of ≥1.32 (fold change ≥2.5) with a false-discovery-rate adjusted *p*-value of less than 0.05. Using this criterion, we determined that the CgrA regulon at the 24 h time point in cyst development is comprised of 258 DEGs (Table 1 and Additional file 1: Table S1). Among these a total of 199 genes were found to be CgrA-activated (eg. expression levels lower in the CgrA deletion strain than in the wild type strain) and 59 were CgrA-repressed (eg. expression higher in the CgrA deletion strain than in the wild type strain) (Fig. 1).

**Table 1:** Genes with an upstream CgrA binding peak and CgrA dependent expression

Peak Number	Adjacent Gene locus	Cyst development gene regulation^1^	Gene Name	Strand	Distance (bp) of peak summit to first codon	Gene/Operon	Function of gene product	Log2 (fold_change)	Predicted CgrA binding sequence
COG C: Energy Production and Conversion									
7	RC1_1074	yes	*coxM*	+		Gene in Operon	carbon monoxide dehydrogenase	−1.81138	CGTTCTCACCTTCACG
7	RC1_1075	yes	*coxL*	−	−605	First Gene in Operon	carbon monoxide dehydrogenase	−1.73844	CGTTCTCACCTTCACG
COG E: Amino Acid Metabolism and Transport									
10	RC1_1588	yes	*alkJ*	+	−258	Single Gene	alcohol dehydrogenase	−1.72171	CGTGACCGCCGTCACA
COG K: Transcription									
14	RC1_2001	yes		+	−723	Single Gene	RNA polymerase sigma-70 factor	−6.61303	CTTTCGATGGCTCTCA
6	RC1_0849	yes		+	−195	Single Gene	transcriptional regulator, LuxR family protein	−2.03622	TGTTGTCGGCATCAAG
15	RC1_2169	yes		−	−92	Single Gene	RNA polymerase sigma-32 factor	−1.55582	CGCAAGGCGCAGCGCC
18	RC1_2981	−	*chrR*	+	−199	Single Gene	transcriptional activator ChrR	1.36715	TGTGATCCATTTTGCG
COG L: Replication and Repair									
13	RC1_1927	−		+	−479	Single Gene	CRISPR-associated protein	−1.37779	TTTGCCCACCCACAGA
24	RC1_3483	−		+		Gene in Operon	CRISPR-associated protein	−3.95202	TCTGAATGATATCAAG
24	RC1_3484	−		+		Gene in Operon	CRISPR-associated RAMP protein	−3.95202	TCTGAATGATATCAAG
24	RC1_3482	−		+	−661	First Gene in Operon	CRISPR-associated protein	−3.68884	TCTGAATGATATCAAG
COG M: Cell wall/Membrane/Envelope biogenesis									
28	RC1_4002	yes	*wcaJ*	−	−174	Single Gene	colanic biosynthesis UDP-glucose lipid carrier transferase	−2.9087	TTTGTGACGCTTAGAA
16	RC1_2533	yes	*exoP*	−	36	Single Gene	succinoglycan biosynthesis transport protein ExoP	−2.20703	CTCGCGGAAGGGAATG
COG N: Cell Motility									
9	RC1_1393	−	*flgI*	+	−270	First Gene in Operon	flagellar P-ring protein FlgI	−2.13001	CCAGATATTGGTAACA
COG Q: Secondary Metabolites Biosynthesis, Transport, and Catabolism									
8	RC1_1261	−	*frnE*	+	−139	Single Gene	DSBA-like thioredoxin family protein	2.02091	CGTCACGTAGGTTTCG
COG R: General Functional Prediction Only									
7	RC1_1072	yes	*coxE*	+		Gene in Operon	CoxE protein	−1.81138	CGTTCTCACCTTCACG
7	RC1_1073	yes	*coxD*	+		Gene in Operon	carbon monoxide dehydrogenase D protein	−1.81138	CGTTCTCACCTTCACG
COG S: Function Unknown									
14	RC1_1997	yes		+		Gene in Operon	R body protein	−9.38585	CTTTCGATGGCTCTCA
14	RC1_1998	yes		+		Gene in Operon	R body protein	−9.35414	CTTTCGATGGCTCTCA
14	RC1_1996	yes		+		Gene in Operon	R body protein	−8.84511	CTTTCGATGGCTCTCA
14	RC1_1994	yes		+		Gene in Operon	R body protein	−8.34157	CTTTCGATGGCTCTCA
14	RC1_1993	yes		+		Gene in Operon	R body protein	−8.21744	CTTTCGATGGCTCTCA
14	RC1_1995	yes		+		Gene in Operon	R body protein	−7.64522	CTTTCGATGGCTCTCA
25	RC1_3786	−	*gcyB*	+		Gene in Operon	hypothetical protein	−6.4452	TGTGAAGCAGTTCACA
25	RC1_3787	−	*gcyC*	+		Gene in Operon	hypothetical protein	−6.08831	TGTGAAGCAGTTCACA
5	RC1_0834	yes		+	−202	Single Gene	hypothetical protein	−2.6779	GATAAAGTGGCGCACA
9	RC1_1394	−	*cheL*	+		Gene in Operon	chemotactic signal-response protein CheL	−2.13001	CCAGATATTGGTAACA
12	RC1_1693	yes		−	−226	Single Gene	phasin family protein	−1.93541	CGCACCGTCCCGCAGA
24	RC1_3478	−		+	−161	Single Gene	hypothetical protein	−1.74953	GGCAAATAACAACAAA
2	RC1_0093	−		+	−204	Single Gene	phage protein Gp37	−1.4638	CGCGAAGATCGGCGCA
COG T: Signal Transduction									
6	RC1_0849	yes		+	−195	Single Gene	transcriptional regulator, LuxR family protein	−2.03622	TGTTGTCGGCATCAAG
20	RC1_3006	−	*pleC*	+	−90	Single Gene	non-motile and phage-resistance protein	1.39601	GGTGCCCCGGTTCTCC
27	RC1_3832	−		−	−118	Single Gene	methyl-accepting chemotaxis protein	2.05864	CTTAAAGGGGGGTAAA
COG V: Defense Mechanisms									
1	RC1_0024	yes		+	−52	Single Gene	ABC transporter ATP-binding protein	−2.84109	GGCGGTCCCGGTCACC
COG not assigned									
25	RC1_3785	−		+	−188	First Gene in Operon	hypothetical protein	−8.90946	TGTGAAGCAGTTCACA
14	RC1_2000	yes		−	−96	First Gene in Operon	hypothetical protein	−6.52071	CTTTCGATGGCTCTCA
25	RC1_3784	−		−	−43	Single Gene	hypothetical protein	−6.24446	TGTGAAGCAGTTCACA
14	RC1_1999	yes		+		Gene in Operon	R body protein	−5.42666	CTTTCGATGGCTCTCA
17	RC1_2730	yes		−	−855	Single Gene	hypothetical protein	−4.97489	TTTGAAGTTCCCCCCA
4	RC1_0632	−		−	−28	Single Gene	hypothetical protein	−3.29529	TGTACAGCACCTCGAA
22	RC1_3373	yes	*ecnAB*	−	−145	Single Gene	ecnAB; entericidin EcnAB family protein	−2.03569	GGTGACCTTCCTGGGG
11	RC1_1636	yes		+	−79	Single Gene	hypothetical protein	−1.47009	CGTGCCGCCCGGAAAG
3	RC1_0163	−		+	−415	Single Gene	hypothetical protein	1.33965	TTCAAAATACCGCGAA
19	RC1_2985	−		+	−651	Single Gene	hypothetical protein	1.5141	CTTGTAGAGCTTCATG
21	RC1_3208	−		+	−158	Single Gene	ABC transporter substrate-binding protein	1.60476	CGCGACGGGGGGAACG
26	RC1_3822	yes		−	−146	Single Gene	putative Ig domain-containing protein	2.87758	TTTGATAAAATCCAAT

^1^Genes that were previously observed to be differentially expressed during cyst development [26]

### RNA-Seq reveals CgrA-regulated genes with an involvement in cyst development

**Fig. 2:**
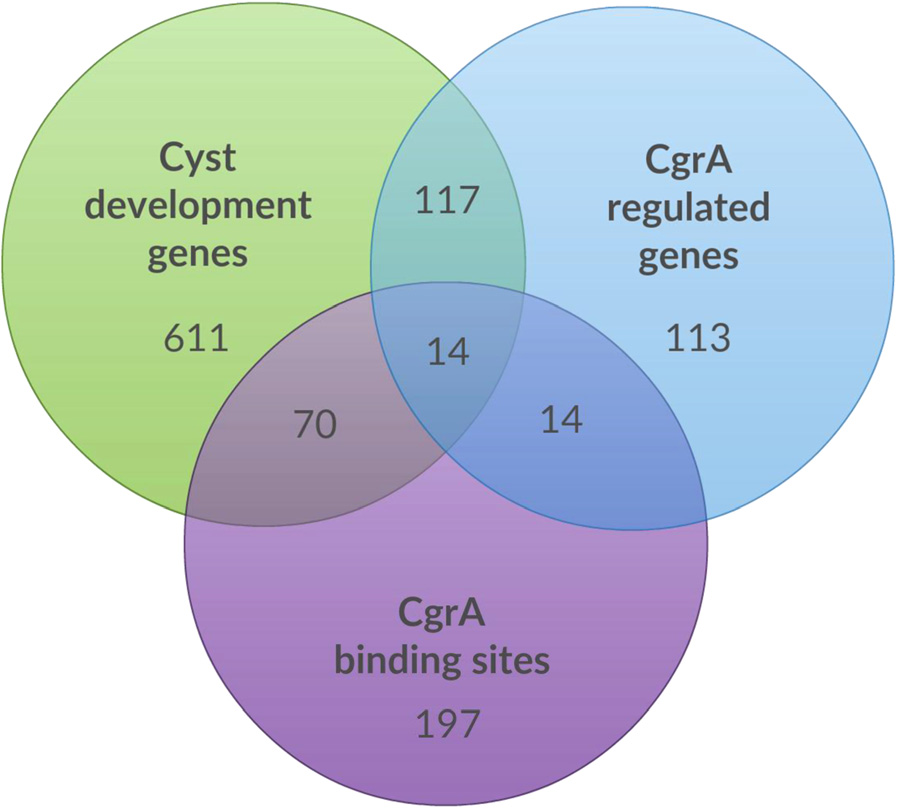
A Venn diagram depicting the number of genes in the CgrA regulon versus genes that undergo differential expression during cyst development. The green circle indicates genes shown to be developmentally regulated during encystment in a previous study [26]. The blue circle are members of the CgrA regulon as based on RNA-seq (blue) and the purple circle are members of the CgrA regulon as based on ChIP-seq analyses

To facilitate an overview of the function of individual loci, we subcategorized each DEG into clusters of orthologous groups (COG) (Fig. 1, Tables 1 and Additional file 1: Table S1). These COG’s designations correlate with a previous study where we categorized genes in similar COG groups that undergo changes in expression as wild type cells differentiated from vegetative into fully developed cysts over a 4 day period [26]. Among the 258 observed DEGs in the CgrA regulon, a total of 131 of these genes were observed in a previous RNA-seq study to be differentially expressed during cyst development (Fig. 2) [26].

As shown previously, cyst cell development involves significant changes in cell membrane and cell wall composition that presumably provides extreme desiccation resistance. As indicated in Tables 1 and Additional file 1: Table S1, members of the CgrA regulon include genes involved in lipid metabolism (COG I), cell wall/membrane/envelope biogenesis (COG M), and carbohydrate metabolism and transport (COG G). Several CgrA regulated genes involved in fatty acid metabolism (COG I), such as acyl-CoA dehydrogenases coded by *RC1_3534* and *RC1_3949,* acetyl CoA acetyltransferase coded by *RC1_3948* were previously shown to undergo an increase in expression as cyst cells develop. However, in the *ΔcgrA* mutant strain, induced expression of these fatty acid metabolism genes were absent, indicating that they are part of the CgrA regulon. In COG M there are also many genes involved in exopolysaccharide (EPS) biosynthesis that we previously reported underwent alterations in expression during cyst cell development [26]. However in the *ΔcgrA* mutant strain, EPS genes including *RC1_3988, RC1_3996, RC1_4000, RC1_2533, RC1_2543, RC1_1410, RC1_2536* and *RC1_2537* no longer exhibited increased expression indicating that these EPS genes are part of the CgrA regulon. Finally, the *ΔcgrA* mutant strain is also defective in regulating expression of numerous membrane protein transporters previously observed to undergo changes in expression during cyst development. This includes; a variety of sugar transporters coded by *RC1_2705, RC1_3730, RC1_3731, RC1_3732, and RC1_3734*; two TonB homologs (*RC1_0278* and *RC1_3723)* and several TonB dependent transporters (*RC1_0403*, *RC1_0945*); an outer membrane efflux protein (*RC1_3821);* a B_12_ transporter *RC1_3736,* and a ferrichrome receptor (*RC1_3892)*. Thus, the CgrA regulon encompasses a large number of genes that affect cyst cell membrane composition, transport and cell wall biosynthesis many of which were known to undergo changes in expression during early cyst differentiation [26].

In addition to genes controlling cell membrane and cell wall biosynthesis, there are other notable members of the CgrA regulon. This includes 20 genes involved in replication and repair (COG L), 18 genes involved in signal transduction (COG T), 12 genes involved in transcription (COG K). Detailed analysis of genes in these additional categories will be discussed below when we describe ChIP-Seq analysis, which allows differentiation between direct and indirectly regulated members of the CgrA regulon.

### Identification of genome-wide CgrA binding sites by ChIP-Seq

We addressed which genes in the CgrA regulon are under direct control of CgrA by performing a genome-wide search for CgrA binding sites. For this analysis we used chromatin immunoprecipitation (ChIP) with a CgrA-3xFLAG construct that allowed us to immunoprecipitate CgrA bound DNA segments. In this assay, chromatin bound CgrA was cross-linked to its target site in vivo, the DNA was then isolated, sheared, depleted of non-specific protein and nucleic acids by immunoprecipitation of CgrA bound segments, de-crosslinked from CgrA and then sequenced by high-throughput DNA sequencing. Genomic DNA taken prior to immunoprecipitation was used as an ‘input’ control.

**Fig. 3:**
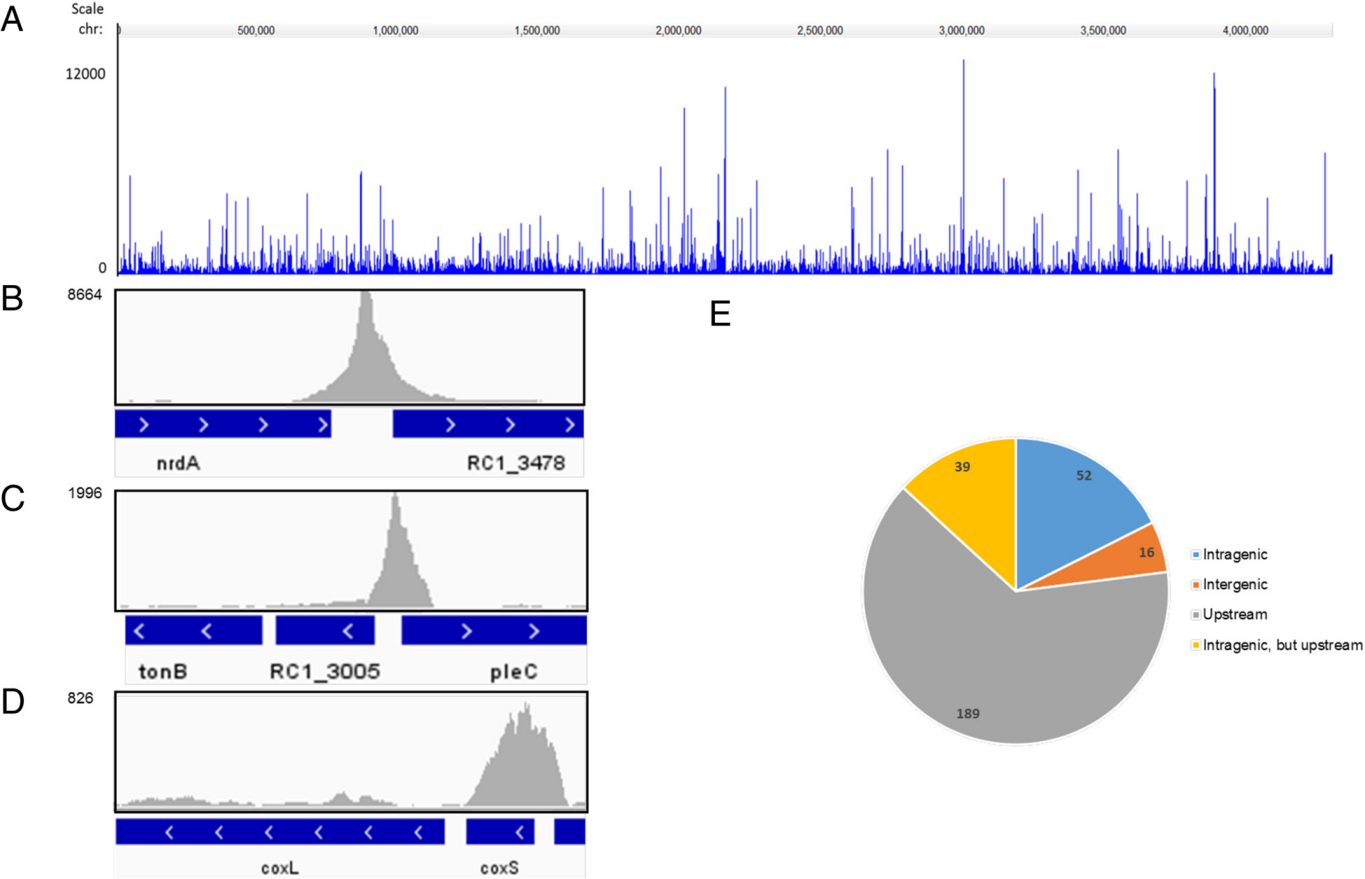
Genome-wide mapping of CgrA binding sites. **a** shows JBrowse view of 295 CgrA binding peaks across the *R. centenum* genome as determined by ChIP-seq. Peak height indicates the sequencing read depth at each genomic position. **b** Representative ChIP-seq peak that mapped to an intergenic locus upstream of a gene. **c** Representative ChIP-seq peak located between divergently transcribed gene pairs. **d** Representative ChIP-seq peak located within a protein coding sequence with a gene downstream of the binding sites. **e** Distribution of the genomic localizations of the binding sites with regard to the closest situated genes. For binding inside a coding sequence, we distinguished between purely intragenic positions (intragenic) and intra genic positions upstream of another transcription unit (Intragenic, but upstream)

We were able to map 97 % of the sequences uniquely to the genome with near-complete representation of the entire genome (98 % of the genome was mapped). To visualize the genome coverage, the sequencing reads were mapped to each position on the genome using Bowtie and the traces were visualized in Jbrowse and IGV. Peaks were called using a model-based analysis (MACS) to identify enriched regions in the CgrA-IP compared to the input control (sheared genomic DNA), with *p* value cutoff =1.00e-05. To validate the results, the data were also analyzed using the MOSAiCS package with peaks discarded if they were called in only one of the two methods. As seen in Fig. 3A, and detailed in Additional file 2: Table S2, 295 peaks denoting CgrA-binding sites were called as significant along the entire genome.

**Fig. 4:**
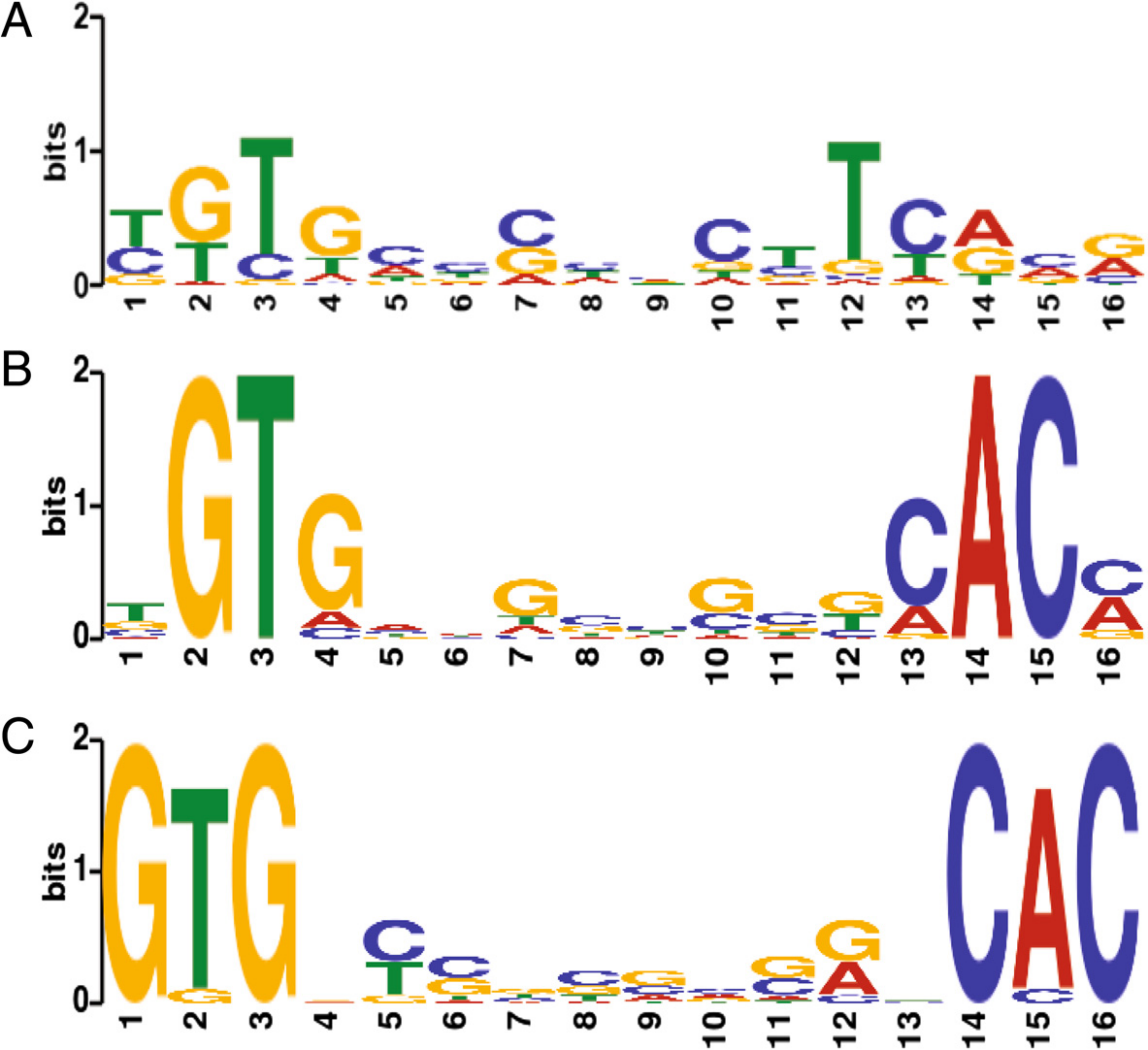
Consensus CgrA binding motif. **a** Consensus binding sequence identified from MEME analysis of 28 ChIP-Seq peaks that also exhibited CgrA dependent changes in gene expression. **b** A related alternative consensus sequence motif identified in 27 additional ChIP-Seq peaks. **c** A related consensus sequence with alternative spacing found in 20 ChIP-Seq peaks

A consensus CgrA binding motif was initially searched among a group of 28 IP peaks that are located adjacent to genes that also exhibit regulation by CgrA as based on RNA-Seq analysis. In a previous study, we used DNAse I protection footprint analysis to characterize a well-defined CgrA binding site located upstream of the *RC1_3785*-*gcyB-gcyC-cgrA* promoter region [10]. A 16 bp palindrome sequence that was protected by CgrA TGTGA-N_6_-TCACA was identical to the reported *E. coli* CRP consensus motif [27, 28]. Consequently, we analyzed these 28 CgrA binding peaks for sequences that resembled this motif using the dynamic programming algorithm FIMO, with the position weight matrix (PWM) of the CRP motif as an input. This analysis showed the existence of closely related sequence derivatives in all 28 analyzed peaks exhibiting a *p* value <0.01. These derivative sequences were subsequently extracted and aligned using motif-based sequence analysis tool MEME to generate a consensus motif exhibiting a low *E* value of 1.2e-029 (Fig. 4A). The consensus sequence derived from these 28 peaks were then used to probe the remaining 267 peaks for the presence of this sequence again using FIMO from which an additional 251 closely related sequences (*p* < 0.01) were obtained (Additional file 2: Table S2). A related alternative sequence motif GTG-N_8_-CAC that is part of the CRP tGTGA-N_6_-TCACa recognition sequence was also found in 27 peaks (Fig. 4B). Finally, a GTG-N_10_-CAC motif with alternative spacing is present in 20 peaks (Fig. 4C), possibly suggesting the existence of a different sub-regulon for this transcription factor.

Inspection of CgrA binding-sites relative to the location of neighboring genes and/or within genes revealed that 69 % of all called binding sites were located at intergenic regions. Since ∼ 90 % of the entire *R. centenum* genome represents intragenic sequences, this means that there is significant enrichment of intergenic over intragenic sequences. The peak height (number of sequence reads) between intragenic and intergenic locations was approximately equal irrespective of the fold-enrichment used as a cutoff, indicating that CgrA binds with similar affinity to both types of sites.

### Identification of directly regulated members of the CgrA regulon

The availability of both the *ΔcgrA t*ranscriptome profile (RNA-seq data) and CgrA binding profile (ChIP-seq) allows us to undertake a correlation study to determine which of these 258 DEGs are directly regulated by CgrA and which are indirectly regulated (Tables 1 and Additional file 1: Table S1). For this analysis, we first distributed the ChIP-Seq identified CgrA binding-sites into different categories. One of the most frequent categories (189 CgrA binding sites) involves a CgrA binding site mapped uniquely to an intergenic region located just upstream of a gene (Fig. 3B). Among this group there are 71 cases where the CgrA-binding site was located between divergently transcribed gene pairs (Fig. 3C). There are also 39 CgrA binding sites located within a protein coding sequence that have a gene located immediately downstream of the binding site (Fig. 3D). Another category include 15 sites that are located in rRNA/tRNA coding regions and 37 sites that map within a protein coding gene without an adjacent downstream gene, indicating a possible role in long-distance regulation and/or chromosome organization (Fig. 3E).

To identify directly regulated members of the CgrA regulon, we cross-examined the ChIP-seq and RNA-seq data sets to identify genes that (i) exhibited CgrA-dependent expression (258 DEGs) and (ii) had a CgrA binding site in their putative promoter regions (−1000 bp to +50 bp relative to gene start codon) (Table 1). Among the 258 genes that have altered expression in the *ΔcgrA* strain, 30 of these are directly downstream of a ChIP-seq identified CgrA binding site (Table 1). 22 out of these 30 genes are down-regulated in the deletion strain and 8 are up regulated, indicating that CgrA is preferably functioning as an activator. Of the 30 genes that are adjacent to a CgrA bound intergenic region, there are two examples of divergent genes that are both regulated by CgrA, correlating to the fact that CgrA directly regulates 30 genes by binding to 28 distinct regions among 295 total binding sites. Specifically, the divergent *RC1_2000* and *RC1_2001* genes are both activated ~12-fold by a CgrA binding site located in their common intergenic region and the other pair is *RC1_3784* and *RC1_3785,* which are activated 12- and 18-fold, respectively by CgrA. A total of 25 singular genes appear to be directly regulated by CgrA. There are also 5 CgrA binding sites upstream of genes that appear to be the first gene of potential operons. Only a few operons in the genome have been characterized in this species, but for the purpose of this analysis, we defined genes to be in an “operon” if they are read in the same direction, are temporally co-regulated to similar extent (as based on similar RNA-seq measured expression levels) and are separated by fewer than 50 bp. When all genes from potential operons are counted, the number of loci that are directly regulated by CgrA expands to 45 genes.

We also cross-referenced the Chip-seq peaks in this study with genes that were shown in a previous study to undergo changes in expression during later times in the cyst developmental cycle (Fig. 2; yellow highlighted genes in Additional file 2: Table S2) [13]. There are 84 ChIP seq peaks next to genes that were shown in our previous RNA-seq study [26] to undergo changes in expression during mid and late cyst development periods. This group includes genes involved in changing the cell membrane composition as well as several additional transcription factors and signal transduction components such as histidine kinases and proteins involve in the synthesis and degradation of di-c-GMP (Additional file 2: Table S2). Members of this category did not exhibit CgrA dependent changes in expression during the 24 h time point that we surveyed with RNA-Seq. However, we assume that this latter category represents genes that are directly regulated by CgrA at a later time point in development most likely in conjunction with other transcription factors. When taking this category into account, the number of directly regulated genes in the CgrA regulon increases to at least 131 total genes.

### CgrA directly controls regulatory genes involved in cyst development

In a previous study, we utilized qRT-PCR to demonstrate that expression of potential *RC1_3785*-*gcyB*-*gcyC* operon that codes for non-catalytic subunits of guanylyl cyclase, was dependent on CgrA [10]. In confirmation of the validity of the RNA Seq data in this study, we also observed a 60- to 80-fold reduction of *gcyB* and *gcyC* expression in the Δ*cgrA* strain (Table 1). There is a ChIP-seq site immediately upstream of the putative *RC1_3785*-*gcyB*-*gcyC-cgrA* gene cluster. These results confirm that expression of several cyclase subunits is dependent on CgrA. Interestingly, divergent to the *RC1_3785* is a small open reading frame *RC1_3784* that also exhibits a 60-fold CgrA dependent increase in expression (Table 1). There has been no reported disruption of *RC1_3784* nor of *RC1_3785,* however the fact that they exhibit similar expression levels as the linked *gcyB*-*gcyC* genes suggests that they may also have a role in cGMP production.

In addition to directly controlling production of cGMP, CgrA also directly controls the expression of several sigma factors. The genome of *R. centenum* contains 19 annotated sigma factors, 7 of which were previously shown to undergo changes in gene expression during cyst development [26]. In this regard, we observed that *RC1_2001* that codes for a σ^70^ and *RC1_2169* that codes for a σ^32^ are both directly regulated (12-fold and 3-fold, respectively) by CgrA. (Table 1) Expression of this σ^70^ (*RC1_2001*) was previously shown to be significantly ramped up early in development similar to that of the guanylyl cyclase subunits coded by *gcyB*-*gcyC* [26]. The expression of the anti-sigma factor gene *chrR* (*RC1_2981*) that is reported to inhibit the activity of the extracytoplasmic function (ECF) sigma-E (RpoE) [29] is also directly repressed by CgrA. Collectively, these results indicate that CgrA regulates cyst formation process in part by controlling the expression of several sigma factors that in turn likely control expression of additional downstream genes in a hierarchical manner.

CgrA also directly regulates the transcription of a solo LuxR family transcription factor coded by *RC1_0849*. LuxR is known as an important regulator in bacterial quorum sensing networks involved in biofilm formation, virulence, cell division and cell wall synthesis in other bacteria [25, 30, 31]. A CgrA binding site is located in the intergenic region between *RC1_0848* (also encoding a solo LuxR transcription factor) and *RC1_0849* with expression of *RC1_0849* down-regulated 4-fold in the *ΔcgrA* mutant (Table 1).

In regards to cell cycle control, CgrA directly regulates the expression of *RC1_3006* that codes for a homolog of the histidine kinase PleC (Table 1). PleC is proposed to regulate the cell cycle in *Caulobacter crescentus*, *Agrobacterium tumefaciens* and *Sinorhizobium meliloti* [32–34]. All of these organisms, including *R. centenum,* are members of the alpha-proteobacteria lineage suggesting that PleC may also have a role in cell cycle control in *R. centenum*.

### ChIP-Seq analysis expands the number of CgrA controlled regulatory elements

Of the 295 ChIP-Seq called CgrA binding sites, a great number are associated with genes coding for proteins involved in transcription and/or signal transduction (Additional file 2: Table S2). For example, CgrA binds upstream of three additional genes coding for sigma subunits (*RC1_0261, RC1_1724* and *RC1_2225*). There is also a CgrA binding site located upstream of *RC1_1407* that codes for the nitrate/nitrite response regulator NarL. This is notable as alteration of the nitrogen content of growth medium is one of several factors that can lead to cyst induction [8]. CgrA binding is also detected upstream of *expG* (*RC1_1346*) which is a transcription factor that is involved in regulating exopolysaccharide synthesis. Exopolysaccharide gene expression is also highly regulated in response to cyst development [26]. There are also CgrA binding sites detected upstream of 12 additional annotated transcription factors (*RC1_0082, RC1_0438, RC1_0494, RC1_0907, RC1_1086, RC1_1536, RC1_1746, RC1_2253, RC1_2513, RC1_2951, RC1_3027* and *RC1_3568).*

In regards to cell cycle progression, we have mentioned above that expression of PleC is affected by deletion of CgrA and that it also has a CgrA binding site located upstream of its coding sequence. However in addition to PleC, cell cycle progression in *Caulobacter* also involves two DNA binding master regulatory proteins CtrA and GcrA [35]. CtrA in *R. centenum* is also known to undergo differential changes in expression as cells transition into cyst form [26] and has been shown to affect cyst cell development [36]. There is no detectable CgrA binding upstream of *ctrA* (*RC1_0209*) however there is detectable binding of CgrA upstream of a GcrA homolog (*RC1_0907*). These results suggest that *R. centenum* may utilize cell cycle regulators similar to that of other alpha-proteobacteria and that CgrA directly regulates several of these components.

In regards to signal transduction, there is CgrA binding detected upstream of a gene coding for the histidine kinase CstS2 (*RC1_2047)*, which is known to be involved in cyst formation [6, 11] as well as histidine kinases coded by *RC1_0633, RC1_0908, RC1_2541* (Additional file 2: Table S2)*.* The binding site located upstream of *RC1_2541* is particularly intriguing as *RC1_2541* codes for a homolog of the histidine kinase RegB that is divergently transcribed from its cognate response regulator RegA (*RC1_2542).* RegB/RegA are global regulators that control such processes as respiration, photosynthesis, carbon fixation, nitrogen fixation, hydrogen uptake, heme and cytochrome synthesis in response to alterations in the redox state of the ubiquinone pool [36–38]. RegB/RegA are best known for their roles in activating synthesis of the photosystem suggesting that CgrA may down-regulate photosystem synthesis. In this regard, it’s also notable that CgrA binding is detected upstream of three operons involved in synthesis of bacteriochlorophyll (*RC1_2078* and *RC1_2093*) and carotenoids (*RC1_2091*) as well as upstream of the AerR-PpsR regulators of the photosystem (Additional file 2: Table S2).

Finally, in addition to controlling synthesis of cGMP, CgrA also appears to have a role in controlling synthesis of a second nucleotide signaling molecule, di-c-GMP. Specifically, CgrA binding is detected upstream of two hybrid diguanylate cyclase/phosphatase coded by *RC1_3168* and *RC1_3358* and upstream of two diguanylate cyclases coded by *RC1_3881* and *RC1_3923* (Additional file 2: Table S2). The expression of the diguanylate cyclases coded by *RC1_3358*, and *RC1_3881* are known to be down-regulated late during cyst development [26].

### Indirectly regulated members of CgrA regulon that contribute to the control of cyst development

Expression of 213 differentially regulated genes appear to be indirectly controlled by CgrA as evidenced by changes in expression coupled in the *ΔcgrA* strain coupled with an absence of a CgrA dependent ChIP-Seq peak (Additional file 1: Table S1). Presumably, these indirectly controlled genes are regulated by secondary regulatory protein(s), specifically, by one or more of the sigma subunits and/or transcription factors that are directly regulated by CgrA. There is also the possibility several indirectly regulated proteins may be regulated at a tertiary level involving secondary regulators that control expression of additional tertiary transcription factors that subsequently affect cyst development (Fig. 4).

Indirectly controlled members of the CgrA regulon contain genes in all COG groupings encompassing genes involved in metabolism, cell membrane and cell wall biosynthesis, and transport the majority of which are known to undergo changes in expression as cells develop into cysts [26] (Additional file 1: Table S1). In addition, there are numerous genes coding for transcription regulators and signal transduction proteins that are indirectly regulated by CgrA. This latter group include eight transcription regulator genes (*RC1_3729, RC1_1406, RC1_3964, RC1_3072, RC1_1428, RC1_1178, RC1_3526* and *RC1_2973*), three histidine kinase genes (*RC1_3999, RC1_2481,* and *RC1_3061*), two response regulators (*RC1_1406* and *RC1_1592*) and an additional diguanylate cyclase (*RC1_1539*). Furthermore, with exception of *RC1_2973,* all of these regulatory genes were previously shown to undergo differential changes in gene expression when wild type cells undergo cyst development [26]. This latter observation suggests that they may also have downstream roles that affect encystment.

## Discussion

We recently undertook detailed analysis of the transcriptome of wild type *R. centenum* as cells undergo development from vegetative into cyst forms [26]. This prior study demonstrated that 815 genes (19.78 % of the genome) underwent changes in expression during a four-day cyst development period. We also previously established that one of the key signals for inducing cyst development involves the synthesis of cGMP, the production of which increases as cells undergo cyst cell maturation [8]. Production of cGMP, and subsequent induction of cyst development, is regulated by the binding of cGMP to the CRP homolog CgrA, which initiates the encystment development cascade [8, 10].

In this study, we have identified both directly and indirectly regulated members of the CgrA regulon using RNA-Seq and ChIP-Seq. The results of this analysis indicate that 45 genes are directly regulated by CgrA while an additional 213 genes are indirectly controlled. The CgrA regulon includes a large number of genes that function in cyst cell wall synthesis and membrane transport, many of which are known to undergo significant changes during cyst development [26]. To a lesser extent, the CgrA regulon also includes genes involved in cell motility, signal transduction, transcription, and in nutrient metabolism (Tables 1 and Additional file 1: Table S1). The involvement of CgrA on cell motility was unexpected as a role of cell motility has not been shown to be involved in cyst development. One possibility is that CgrA may respond to nutritional stress by bifurcating subpopulations of cells into those that undergo “flight” in the hope of migrating into areas where there are more optimal nutrient opportunities versus cells that undergo encystment for more long-term survival.

A large number of genes that this study defines as members of the CgrA regulon (201 members as identified by ChIP-Seq and/or RNA-Seq) are also congruent with our previous RNA-seq study which identified genes that undergo significant changes in expression during cyst cell development (Fig. 2, Additional file 3: Table S3). Our earlier study was centered on changes in expression that occurred at multiple time points over a 4 day period from early to late cyst development phases, so complete congruence with this analysis that is focused on a defined 24 h time point, is not expected. Furthermore, in nearly all of the cases where CgrA functions as an activator or a repressor of expression there is congruence in whether the same set of genes undergo an increase or a decrease in expression during development of cysts in wild type cells (Additional file 3: Table S3). Indeed the significant overlap that exists between these independent analyses further solidifies the identification of loci that are important for the development of cysts and highlights the critical role that CgrA and cGMP play in regulating induction of encystment.

**Fig. 5:**
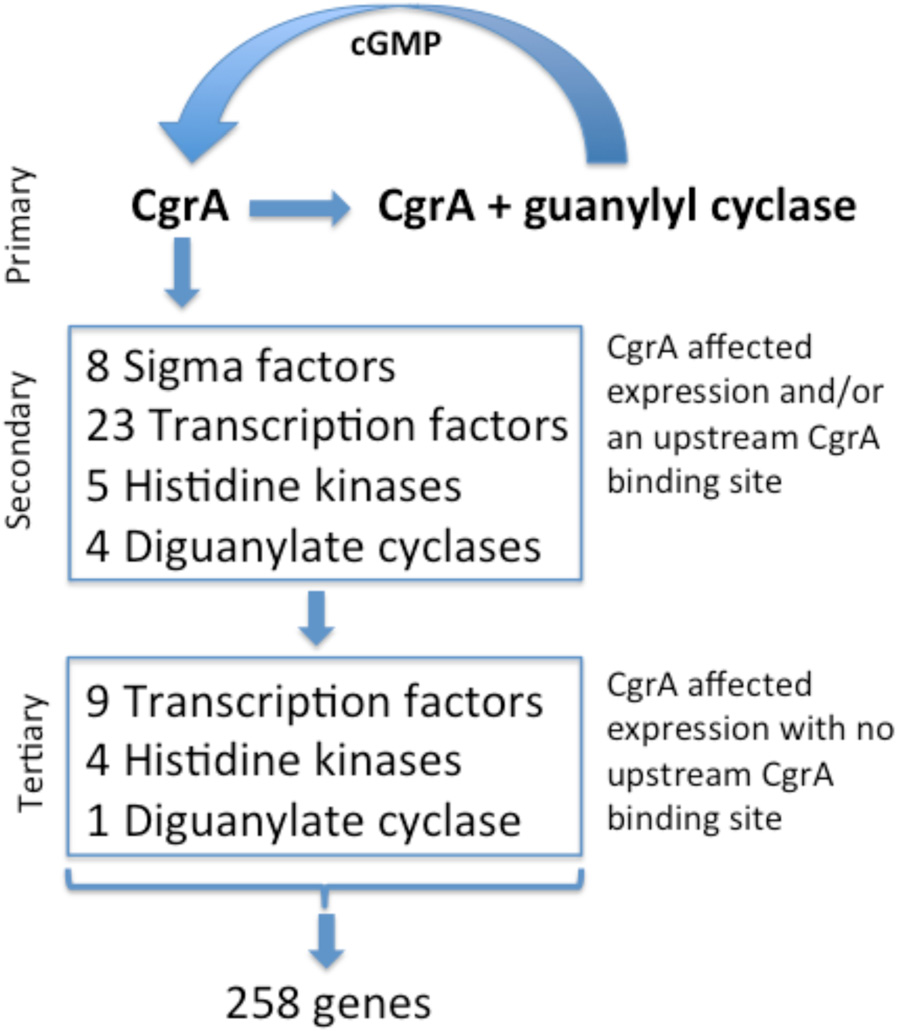
A summary of primary, secondary and tertiary regulators that are part of the CgrA regulon

Important members of the CgrA regulon include genes directly controlled by CgrA that are predicted to be transcription factors or regulators of transcription factors. This includes several sigma subunits, an anti-sigma factor (ChrR; RC1_2981), numerous transcription factors and numerous signal transduction components (Fig. 5). These regulatory genes likely control an additional layer of downstream regulators, such as the 7 transcription factors and 5 signal transduction regulators (histidine kinases and response regulators) that exhibit differential changes in gene expression without nearby CgrA binding sites (Fig. 5). There are many complexities in this plethora of downstream regulators that need to be unraveled. For example, the CgrA-regulated LuxR family transcription regulator coded by *RC1_0849* has a LuxR-type DNA-binding HTH domain linked to response regulator receiver domains. This suggests that its DNA binding activity is likely controlled via phosphorylation by a histidine sensor kinase. However the identity of which histidine kinase may be involved in controlling the activity of this (or other regulators) remains unresolved.

*R. centenum* has 19 annotated sigma subunits of RNA polymerase that are coded by its genome [36]. Six σ^70^ and one σ^32^ are differentially expressed at different times during encystment [26], indicating that the control of encystment gene expression likely involves temporal expression of alternative sigma factors. This is not unlike the development of endospores in Gram-positive bacteria that uses several sigma factors to control the timing of endospore gene expression [37, 38]. The σ^70^ coded by *RC1_2001* is of particular interest as it has a well-defined CgrA Chip-seq peak and its CgrA dependent expression is significantly increased early during cyst development [26]. Furthermore, a mini-Tn5 disruption of this σ^70^ is known to lead to loss of encystment indicating that it likely has a significant role in regulating the cyst development pathway [8].

Another interesting aspect of this study is that a large number of the ChIP-Seq mapped CgrA binding sites did not affect neighboring gene expression (Fig. 2; white sections of Additional file 2: Table S2). This suggests that there may be undefined conditions, such as different times in the developmental pathway, where these silent CgrA binding sites may become important. Indeed it’s quite possible that CgrA binding to these “silent sites” may affect the binding of other transcription factors that have roles at later stages of development, which are not analyzed in our study. Such a scenario would require a much more in depth analysis of the effect of a CgrA deletion at different time points in development, which is beyond the scope of this initial analysis of the CgrA regulon. Similar ChIP-seq studies have also demonstrated that the *E. coli* CRP-cAMP complex not only binds to intergenic CRP sites where it partakes in classic gene regulation [39], but in addition, binds to many silent intragenic sites where it is thought to fulfill a role as chromosome organizer or as a nucleoid associated protein (NAP) [40]. A similar result was reported for CRP at several *E. coli* promoters that have high-affinity DNA-binding sites any yet do not exhibit CRP-dependent regulation [40]. A role as chromosome organizer role has also been proposed for GlxR, a CRP family member from *Corynebacterium glutamicum* that binds to over 100 sites on the genome [41]. Furthermore, recent research has shown that a CRP homolog from *Mycobacterium tuberculosis* can bind to 191 sites where it directly and indirectly regulates the expression of 865 genes [42]. Additionally, ChIP-chip and ChIP-seq analyses of other types of transcription factors in *Rhodobacter capsulatus,E. coli, Salmonella,* and *Pseudomonas* have also identified many transcription factor binding sites that do not correlate with changes in gene expression with corresponding transcriptome experiments [17, 23, 42, 43]. These results raise the possibility of a blurred line between sites where transcription factors function in controlling gene expression and those where they function as NAPs. Clearly these next generation transcriptome studies are revealing a level of complexity of prokaryotic transcription factor binding that extends well beyond that of controlling neighboring gene expression.

## Methods

### Bacterial strains, media, and growth conditions

Wild type *R. centenum* ATCC 51521 and a *cgrA* deletion derivative were cultured aerobically in nutrient rich vegetative CENS or in cyst inducing minimal CENBA medium containing 20 mM butyrate as a carbon source [4, 5]. Liquid grown cells were incubated with shaking in an Erlenmeyer flask at 37 °C or on grown in agar-solidified media at 42 °C.

### RNA isolation

Wild type and *cgrA* deletion strains were grown aerobically overnight to stationary phase in CENS medium at 37 °C. The cells were induced for encystment by sub-culturing into CENBA medium as a 1:50 inoculum dilution. 10 ml samples were collected from three biological replicates at the 24 h period and stored at -80C as a cell pellet. For RNA isolation, cell pellets were thawed, resuspended in RNApro™ Solution (MP Biomedicals) and subsequently disrupted by homogenization using the FastPrep® Instrument (MP Biomedicals) with the Lysing Matrix B setting in impact-resistant 2 mL tubes. Total RNA samples were then extracted using FastRNA® PRO BLUE KIT (MP Biomedicals). For further cleaning, each total RNA sample was treated with RNeasy Mini Kit (Qiagen) following the manufacturer’s RNA clean-up protocol and eluted with 50 μl RNase-free water. Genomic DNA was then removed by addition of 1.5 μl of TURBO DNase (2 Unit/ μl) and 6 μl of 10x TURBO DNase Buffer to 50 μl of RNA sample and then incubated at 37 °C for 30 min. After DNase treatment, RNA clean-up was carried out again with the RNeasy Mini Kit (Qiagen) to remove any remaining protein/DNA contamination. Final RNA concentrations were measured by NanoDrop (Thermo Scientific) with a typical OD_260_ to OD_280_ ratio of RNA samples at approximately 2.0. Final quantification and quality control of total RNA samples were performed using a 2100 Bioanalyzer (Agilent Technologies).

### mRNA transcript isolation, mRNA library construction and sequencing

Library construction and deep sequencing of the library were performed by the University of Wisconsin-Madison Biotechnology Center DNA Sequencing Facility. Briefly, total RNA was reduced of ribosomal RNA content using an EpiCentre® Ribo-Zero™ Magnetic (Bacteria) kit with a targeted 2 μg total RNA input. Illumina mRNA-Seq libraries were prepared from rRNA-depleted samples using the TruSeq™ RNA Sample Prep kit (Illumina, San Diego, CA) per the manufacturer’s protocol. Adapters containing 6 nucleotide indexes were ligated to the double-stranded cDNA with cDNA libraries then amplified with 11 PCR cycles. Single end sequencing (1x100bp) was performed on the Illumina HiSeq 2000 according to the standard Illumina protocol. The resulting sequences were subsequently deposited in the National Center for Biotechnology Information’s Sequence Read Archive (accession no. SRP045612).

### RNA-Seq data analysis

RNA-seq data analysis was carried out on GALAXY platform (http://galaxyproject.org/) [44, 45]. Sequences of 100-nt in length were first trimmed using FASTQ Quality Trimmer (version 1.0.0) with a window size of 6, step size of 6 and quality score greater than 25. Trimmed sequences were mapped to the annotated 4,355,548 base pair *R. centenum* ATCC 51521 genome harboring 4,105 genes using the program Bowtie (version 1.1.2). Transcripts were assembled with Reads per Kilobase of Transcripts Mapped (RPKM) values calculated using Cufflinks (v2.1.1). Mapped RNA-Seq reads were visualized using the Integrative Genomics Viewer. Fold-change calculations were undertaken between wild type and *cgrA* deletion strain samples at 24 h in CENBA medium using Cuffdiff with geometric library normalization method and minimum alignment counts of 10. Genes were considered to exhibit significant differential changes in expression (DEG) when log_2_ of fold change was ≥1.32 with a false discovery rate adjusted *p* value of <0.05. Orthologous groups of DEGs were subgrouped into classifications according to eggNOG (evolutionary genealogy of genes: Non-supervised Orthologous Groups, version 4.0, http://eggnog.embl.de/version_4.0.beta/).

### ChIP-Seq identification of CgrA binding sites

An ectopically expressed CgrA-3xFLAG construct (pSRC2) was constructed and used as bait to enrich for DNA segments that contain bound CgrA. The CgrA-FLAG plasmid construct was made by fusing 3 tandem FLAG epitopes (5′ DYKDHDGDYKDHDIDYKDDDDK-3′) following a short AGSAAGSG spacer.

For construction, a forward primer PcgrEF (aggagggatccTGGAGGACGATGACGAGGTCGC) was used along with the reverse primer PcgrEFlagLrev (ctcctgaattcctacttgtcatcgtcatccttgtagtcgatgtcatgatctttataatcaccgtcatggtctttgtagtcgccagaaccagcagcggagccagcGCCGGCGAGCTTCTCCAGATA) with the resulting PCR segment cloned into pBBR1:MCS-5 to yield pSRC2. The resulting clone was verified by restriction digestion analyses and sequencing. pSRC2 was delivered into Δ*cgrA* strain by conjugation with transconjugants selected on CENS Gm10 media. Complementation of the Δ*cgrA* strain defect in encystment phenotype was shown by growth on CENS Gm10 media containing 200 uM cGMP with cyst development observed microscopically. The expression level of CgrA 3XFLAG was also assayed by Western blot analysis of mid-exponential phase cultures of wild-type *R. centenum* and pSRC2 strains per manufactures instructions (Amersham ECL Prime Western blotting detection reagents; GE Healthcare).

For ChIP-seq analysis 100 ml culture of a *ΔcgrA* strain containing pSRC2 was grown in CENS medium to late log phase at which time all culture was washed and resuspended in CENBA media and incubated for an additional 30 min. In vivo cross-linking of nucleoprotein complexes was then performed by treating cells with formaldehyde to a final concentration of 1 %. After 20 min incubation at 22 °C, cross-linking was quenched by the addition of glycine to 125 mM. Cells were then harvested from 100 ml culture by centrifugation for 10 min at 5,000x*g*, washed twice with Tris-buffered saline (20 mM Tris-HCl pH 7.5, 150 mM NaCl), re-suspended in 4 ml of ice-cold FA-M2 buffer (50 mM Tris-HCl pH 7.5, 150 mM NaCl, 1 mM EDTA, 1 % Triton X-100) and passed through French press twice at 18,000 psi. Following lysis, cellular DNA was sheared by sonication to an average size of 150 to 500 bp. Cellular debris was removed by centrifugation for 10 min at 15000x*g* and the supernatant was retained for use as the input control in immunoprecipitation (IP) experiments.

A 4 ml supernatant of the input sample was incubated with 70 μl ANTI-FLAG® M2 Affinity Gel (Sigma-Aldrich) overnight at 4 °C on a rotating wheel followed by washing three times in cold TBS. An immunoprecipitation experiment without antibody was also set up as a negative control. 150 ng/μl of 3X FLAG peptide was used to elute CgrA-linker-3X FLAG protein for 30 min at 4 °C. Eluted supernatants were then de-crosslinked by incubation overnight at 65 °C with 10 ng/μl Proteinase K. DNA fragments were purified by ethanol precipitation and resuspended in 50ul TE. DNA sample size and concentration were analyzed using Agilent 2100 Bioanalyzer.

Library construction and sequencing of DNA fragments ranging between 150 to 250 bp were performed by the University of Wisconsin-Madison Biotechnology Center DNA Sequencing Facility. Sample library was constructed according to Bioo Scientific’s NEXTflex Chip-Seq Kit protocol with 11 PCR amplification cycles. Single end sequencing (1x100bp) was performed on the Illumina HiSeq 2000 according to the standard Illumina protocol. The resulting sequences were subsequently deposited in the National Center for Biotechnology Information’s Sequence Read Archive (accession no. SRP045612).

### ChIP-seq data analysis

Sequences of 100-nt in length were first trimmed using FASTQ Quality Trimmer (version 1.0.0) with a window size of 4, step size of 1 and quality score greater than 30. Trimmed sequences were mapped to the annotated *R. centenum* ATCC 51521 genome harboring 4,355,548 base pairs (4,105 genes) using the program Bowtie (version 1.1.2). Mapped sequences were visualized by JBrowse and Integrative Genomics Viewer (IGV) [46]. Peaks were called utilizing MACS with the following parameters: band width = 300, model fold = 10, 30, *p* value cutoff =1.00e-05 to identify enriched regions in the CgrA-IP compared to the input control (sheared genomic DNA) [47, 48]. To further validate our MACS result that applies Poisson distribution to process the data, we also used MOSAiCS which uses negative binominal distribution to call peaks. MOSAiCS also introduce a background model that can take into account GC content bias, which is usefull as *R. centenum* has a very high 70.5 % GC content [49–50]. Peaks that were called by both MACS and MOSAiCS were retained.

### Availability of supporting data

All supporting RNA-seq and ChIP-seq data files were generated from sequences that have been deposited at the National Center for Biotechnology Information’s Sequence Read Archive [ref 51 and 52, respectively] as described in the Methods section.

## Conclusions

*R. centenum* has developed into an excellent model microorganism for studying the development of dormant desiccation resistant cysts by a Gram-negative bacterium. A prior transcriptome study has shown large changes in gene expression during cyst development which include the alteration of expression of many genes involved in membrane and cell wall biosynthesis and the reduction of expression of many DNA replication and protein synthesis genes [26]. One of the key regulatory signals that induces cyst formation is the involvement of cGMP which is induced when cells are challenged with nutrient depravation [8, 10]. cGMP production is subsequently sensed by the CRP-like transcription factor CgrA which subsequently regulates a complex cascade of cyst development genes including genes involved in cGMP production. CgrA thus has a central role in regulating the induction and timing of cyst developmental gene expression. The RNA-seq and ChIP-seq results presented in this study establish which genes constitute members of the CgrA regulon and further show which genes are directly versus indirectly regulated by CgrA containing bound cGMP. These results establish that CgrA directly regulates the expression of a large number of regulatory proteins (8 sigma factors, 23 DNA binding proteins, 5 histidine kinases and 4 diguanylate cyclases) which in turn regulate the expression of additional cyst developmental genes. The CgrA + cGMP complex thus appears to constitute a master regulatory signal for the control of cyst development.

## Additional files

Additional file 1:
**Table S1.** A table of all genes that are indirectly up and down regulated by CgrA. (PDF 159 kb) Additional file 2:
**Table S2.** A table of all called CgrA ChIP-seq peaks and their locations on the genome. (PDF 286 kb) Additional file 3:
**Table S3.** A table that compares the wild type-ΔCgrA RNA-seq results from this study with similar RNA –seq results of wild type cells as they transition from vegetative to cyst forms as obtained by Dong and Bauer [26]. (XLSX 157 kb)
