# Supplementary material for: Mapping the CgrA regulon of Rhodospirillum centenum reveals a hierarchal network controlling Gram-negative cyst development
Source: BMC Genomics. 2015 Dec 16;16:1066. doi: 10.1186/s12864-015-2248-z (PMC4681086; doi:10.1186/s12864-015-2248-z)
Supplement: Additional file 1: — Table S1. A table of all genes that are indirectly up and down regulated by CgrA. (PDF 159 kb) [file 12864_2015_2248_MOESM1_ESM.pdf]

## Table S1: Genes indirectly controlled by CgrA

| Gene locus | Cyst development gene regulation | Gene name | Gene function | log2(fold_change) |
|---|---|---|---|---|
| **COG C: Energy Production and Conversion** | | | | |
| RC1_0780 | yes | coxB | Cytochrome c oxidase subunit II | -2.40495 |
| RC1_0781 | yes | coxA | cytochrome c oxidase subunit I | -1.73419 |
| RC1_1753 | - | | pyridine nucleotide-disulfide oxidoreductase | 2.07987 |
| **COG E: Amino Acid Metabolism and Transport** | | | | |
| RC1_0557 | - | rocD | ornithine aminotransferase | -1.77643 |
| RC1_0762 | yes | ald | alanine dehydrogenase | -1.73455 |
| RC1_2162 | - | ggt | gamma-glutamyltransferase | 2.02528 |
| **COG F: Nucleotide Metabolism and Transport** | | | | |
| RC1_2002 | yes | thyA | thymidylate synthase | -1.46382 |
| **COG G: Carbohydrate Metabolism and Transport** | | | | |
| RC1_0396 | yes | | chitinase class II protein | -5.76675 |
| RC1_3992 | yes | | NAD dependent epimerase | -3.97139 |
| RC1_3991 | yes | | UDP-glucose 4-epimerase | -3.44631 |
| RC1_3732 | yes | malK | maltose | -2.26949 |
| RC1_3887 | yes | gcd | quinoprotein glucose dehydrogenase | -2.054 |
| RC1_3731 | yes | malG | maltose transport system permease malG | -1.98964 |
| RC1_3730 | yes | malF | maltose transport system permease malF | -1.96044 |
| RC1_3734 | yes | | sugar ABC transporter periplasmic sugar-binding protein | -1.93453 |
| RC1_2705 | yes | lamB | maltoporin | -1.54149 |
| **COG H: Coenzyme Metabolism** | | | | |
| RC1_2714 | - | ahcY | S-adenosyl-L-homocysteine hydrolase | -1.67193 |
| RC1_3685 | - | mopB | Molybdenum-pterin binding protein MopB | -1.40746 |
| RC1_3176 | - | hemN | oxygen-independent coproporphyrinogen III oxidase | 1.47599 |
| **COG I: Lipid Metabolism** | | | | |
| RC1_3949 | yes | phbB | acetoacetyl-CoA reductase | -2.69034 |
| RC1_0149 | yes | | AMP-dependent synthetase and ligase | -2.39664 |
| RC1_3948 | yes | | acetyl-CoA acetyltransferase | -1.69195 |
| RC1_3533 | yes | | acetyl-CoA carboxylase carboxyltransferase | -1.46508 |
| RC1_3534 | yes | | acyl-CoA dehydrogenase | -1.46508 |

**COG J: Translation**

| | | | | |
|---|---|---|---|---|
| RC1_2153 | - | rpmJ | 50S ribosomal protein L36 | -1.5342 |
| RC1_3199 | yes | | ribonuclease D | -1.3521 |

**COG K: Transcription**

| | | | | |
|---|---|---|---|---|
| RC1_3729 | yes | | transcriptional regulator, Cro | -2.30044 |
| RC1_1406 | - | narP | nitrate/nitrite response regulator protein narP | -2.06959 |
| RC1_3964 | yes | | hypothetical protein | -1.37481 |
| RC1_3072 | yes | | transcriptional regulator, MarR family protein | -1.36705 |
| RC1_1428 | yes | | transcriptional regulator, PadR family protein | 1.34441 |
| RC1_1178 | - | | transcriptional regulator | 1.7039 |
| RC1_3526 | yes | | transcriptional regulator, TetR family protein | 1.99367 |
| RC1_2973 | - | | DNA-binding protein | 2.96137 |

**COG L: Replication and Repair**

| | | | | |
|---|---|---|---|---|
| RC1_1992 | yes | | transposase | -6.21722 |
| RC1_2950 | - | | transposase, is4 family | -3.90939 |
| RC1_3485 | - | | hypothetical protein | -3.66643 |
| RC1_3486 | - | | CRISPR-associated protein | -3.66643 |
| RC1_3335 | - | | CRISPR-associated protein | -3.13015 |
| RC1_3336 | - | | CRISPR-associated protein | -3.13015 |
| RC1_3337 | - | | CRISPR-associated protein | -3.13015 |
| RC1_3338 | - | | CRISPR-associated protein, CT1973 family | -3.13015 |
| RC1_3339 | - | | CRISPR-associated protein, CT1972 family | -3.13015 |
| RC1_3487 | yes | | CRISPR-associated protein | -3.07955 |
| RC1_1925 | - | | CRISPR-associatedhD domain-containing protein | -1.37779 |
| RC1_3481 | - | | hypothetical protein | -1.3457 |
| RC1_0647 | - | lig | ATP dependent DNA ligase domain-containing protein | 1.4743 |
| RC1_2601 | - | | hypothetical protein | 1.62612 |
| RC1_1303 | yes | recJ | ssDNA-specific exonuclease RecJ | 1.75788 |
| RC1_2852 | - | alkB | alkylated DNA repair protein | 2.66854 |

**COG M: Cell wall/Membrane/Envelope biogenesis**

| | | | | |
|---|---|---|---|---|
| RC1_3992 | yes | | NAD dependent epimerase | -3.97139 |
| RC1_3988 | yes | rfbF | glucose-1-phosphate cytidylyltransferase | -3.73468 |
| RC1_3991 | yes | | UDP-glucose 4-epimerase | -3.44631 |
| RC1_0846 | - | ompA | OmpA family protein | -3.32922 |
| RC1_2543 | yes | rfbP | undecaprenyl-phosphate galactose phosphotransferase | -3.29962 |
| RC1_3996 | yes | rfbF | glucose-1-phosphate cytidylyltransferase | -3.15266 |
| RC1_1410 | yes | | polysaccharide biosynthesis | -3.10323 |

| | | | | |
|---|---|---|---|---|
| RC1_3989 | yes | | polysaccharide biosynthesis protein | -2.46707 |
| RC1_3994 | yes | | glycosyl transferase family protein | -2.35468 |
| RC1_0527 | yes | rfbE | CDP-paratose 2-epimerase | -2.26917 |
| RC1_2571 | yes | | glycosyl transferase family protein | -2.2058 |
| RC1_4000 | yes | | polysaccharide biosynthesis protein | -1.88791 |
| RC1_0526 | yes | galE | UDP-glucose 4-epimerase | -1.65928 |
| RC1_0740 | yes | | heat resistant agglutinin 1 | -1.45721 |
| RC1_2536 | yes | | glycoside hydrolase family protein | -1.39421 |
| RC1_2537 | yes | | glycoside hydrolase family protein | -1.39421 |
| RC1_3821 | yes | | Outer membrane efflux protein | 2.68808 |

**COG N: Cell Motility**

| | | | | |
|---|---|---|---|---|
| RC1_1388 | - | flgG | flagellar basal body rod protein FlgG | -5.03273 |
| RC1_1387 | - | flgF | flagellar basal-body rod protein FlgF | -4.2926 |
| RC1_1397 | - | fliK | flagellar hook-length control protein FliK, putative | -4.24408 |
| RC1_1389 | - | flgA | flagellar basal body P-ring biosynthesis protein FlgA | -3.97722 |
| RC1_3758 | yes | flgE | flagellar hook protein FlgE | -3.87832 |
| RC1_1390 | - | flgH | flagellar L-ring protein FlgH | -3.21149 |
| RC1_3762 | - | flgL | flagellar hook-associated protein 3 FlgL | -2.35857 |
| RC1_1398 | - | flgD | basal-body rod modification protein FlgD | -2.33239 |
| RC1_3761 | - | flgK | flagellar hook-associated protein FlgK | -2.18246 |
| RC1_1008 | yes | motB | chemotaxis MotB protein | 2.01354 |
| RC1_1009 | yes | | chemotaxis protein MotA | 2.01354 |

**COG P: Inorganic Ion Transport and Metabolism**

| | | | | |
|---|---|---|---|---|
| RC1_0403 | yes | | TonB-dependent receptor | -4.63442 |
| RC1_3723 | yes | | TonB-dependent receptor | -4.34201 |
| RC1_0026 | yes | | phosphodiesterase | -3.33394 |
| RC1_0278 | yes | | TonB-dependent receptor | -3.29166 |
| RC1_3892 | - | fcuA | ferrichrome receptor FcuA | 1.62684 |
| RC1_0945 | - | | TonB-dependent siderophore receptor, putative | 1.76022 |
| RC1_3736 | yes | btuB | vitamin B12 transporter btuB | 3.47517 |

**COG Q: Secondary Metabolites Biosynthesis, Transport, and Catabolism**

| | | | | |
|---|---|---|---|---|
| RC1_3997 | yes | | methyltransferase | -3.15266 |
| RC1_3984 | yes | | NDP-hexose 3-C-methyltransferase | -2.08819 |

**COG R: General Functional Prediction Only**

| | | | | |
|---|---|---|---|---|
| RC1_3220 | yes | | sodium/solute symporter | -3.26071 |
| RC1_3986 | yes | | hypothetical protein | -2.94841 |

| | | | | |
|---|---|---|---|---|
| RC1_1409 | yes | | hypothetical protein | -2.26183 |
| RC1_0025 | yes | | GNAT family acetyltransferase | -2.16317 |
| RC1_1414 | yes | | ATPase | -1.47779 |
| RC1_0276 | yes | | hydrolase, alpha | 1.32966 |
| RC1_2310 | - | | aminopeptidase family M28 | 1.43123 |
| RC1_2309 | - | | aminopeptidase family M28 | 1.44742 |
| RC1_0629 | - | | putative sulfite oxidase subunit YedY | 1.64275 |
| RC1_2771 | - | acyII | penicillin acylase II | 1.79987 |
| RC1_3818 | - | | hypothetical protein | 2.0607 |
| RC1_3035 | yes | | ABC-type branched-chain amino acid binding protein | 2.19431 |
| RC1_2763 | yes | | alpha/beta hydrolase superfamily protein | 2.49234 |
| RC1_3374 | yes | | hypothetical protein | 3.09598 |
| RC1_3525 | - | bioH | carboxylesterase bioH | 3.40781 |

**COG S: Function Unknown**

| | | | | |
|---|---|---|---|---|
| RC1_1990 | yes | | hypothetical protein | -6.73466 |
| RC1_1666 | yes | | hypothetical protein | -3.90807 |
| RC1_2622 | yes | | hypothetical protein | -3.75902 |
| RC1_0444 | yes | | hypothetical protein | -3.70565 |
| RC1_1631 | - | | hypothetical protein | -3.41238 |
| RC1_2120 | yes | | hypothetical protein | -3.27333 |
| RC1_3219 | - | | hypothetical protein | -3.26421 |
| RC1_3288 | - | | hypothetical protein | -3.09394 |
| RC1_0021 | yes | | hypothetical protein | -2.99588 |
| RC1_2937 | - | | hypothetical protein | -2.79218 |
| RC1_0022 | yes | | hypothetical protein | -2.78927 |
| RC1_0965 | - | | hypothetical protein | -2.70762 |
| RC1_3995 | yes | | hypothetical protein | -2.70696 |
| RC1_0797 | yes | | hypothetical protein | -2.48192 |
| RC1_0389 | - | | hypothetical protein | -2.38165 |
| RC1_2394 | yes | | hypothetical protein | -2.37215 |
| RC1_0739 | yes | | hypothetical protein | -2.18906 |
| RC1_1599 | yes | ymgE | transglycosylase | -2.17327 |
| RC1_2168 | yes | | hypothetical protein | -2.13324 |
| RC1_0296 | yes | | hypothetical protein | -2.03465 |
| RC1_1895 | - | | phasin family protein | -1.97875 |
| RC1_3312 | - | | hypothetical protein | -1.97769 |
| RC1_2574 | yes | | hypothetical protein | -1.91303 |
| RC1_0811 | yes | | hypothetical protein | -1.90438 |
| RC1_3982 | yes | | hypothetical protein | -1.89931 |
| RC1_2846 | - | | hypothetical protein | -1.81942 |

| | | | | |
|---|---|---|---|---|
| RC1_3307 | yes | | integrins alpha chain | -1.73076 |
| RC1_3446 | yes | | hypothetical protein | -1.64399 |
| RC1_2573 | yes | | hypothetical protein | -1.61939 |
| RC1_3445 | yes | | hypothetical protein | -1.59015 |
| RC1_2530 | yes | | hypothetical protein | -1.58154 |
| RC1_3571 | - | | hypothetical protein | -1.5391 |
| RC1_1193 | - | | glyoxalase | -1.5079 |
| RC1_3782 | - | | hypothetical protein | -1.50031 |
| RC1_0810 | yes | | hypothetical protein | -1.46296 |
| RC1_2535 | - | gumK | glucuronosyltransferase | -1.39421 |
| RC1_1926 | - | | hypothetical protein | -1.37779 |
| RC1_0094 | - | | hypothetical protein | -1.36191 |
| RC1_1010 | - | | hypothetical protein | 1.32687 |
| RC1_0011 | yes | | hypothetical protein | 1.44521 |
| RC1_3936 | yes | | pentapeptide repeat-containing protein | 1.47727 |
| RC1_3894 | - | | PepSY-associated TM helix family protein | 1.71009 |
| RC1_0946 | - | | PepSY-associated membrane protein | 1.76022 |
| RC1_1665 | - | | HAMP domain protein | 2.02279 |
| RC1_2164 | yes | | pentapeptide repeat-containing protein | 2.05144 |
| RC1_3820 | yes | | hypothetical protein | 2.68808 |
| RC1_3819 | - | | hypothetical protein | 2.85186 |
| RC1_2480 | yes | | hypothetical protein | 3.16357 |

**COG T: Signal Transduction**

| | | | | |
|---|---|---|---|---|
| RC1_2697 | - | | methyl-accepting chemotaxis protein | -5.10554 |
| RC1_1539 | - | | diguanylate cyclase | -4.52132 |
| RC1_2481 | - | barA | signal transduction histidine-protein kinase BarA | -3.43028 |
| RC1_3999 | yes | | signal transduction histidine kinase | -2.48227 |
| RC1_2174 | yes | | methyl-accepting chemotaxis protein | -2.35626 |
| RC1_1592 | yes | | response regulator receiver domain-containing protein | -2.14718 |
| RC1_1406 | - | narP | nitrate/nitrite response regulator protein narP | -2.06959 |
| RC1_3221 | yes | | nucleotidyltransferase | -1.95064 |
| RC1_3061 | - | | sensory transduction histidine kinase | -1.92664 |
| RC1_1173 | yes | kaiC | circadian clock protein kinase KaiC | -1.89631 |
| RC1_4093 | - | | methyl-accepting chemotaxis protein | -1.55478 |
| RC1_1956 | - | | universal stress protein family | 1.45325 |
| RC1_2035 | - | | methyl-accepting chemotaxis protein | 1.46346 |
| RC1_3024 | yes | | methyl-accepting chemotaxis protein | 1.80489 |
| RC1_3890 | - | aer | aerotaxis sensor receptor Aer | 2.70833 |

**COG U: Intracellular Trafficking and Secretion**

| | | | | |
|---|---|---|---|---|
| RC1_2734 | yes | | HlyD family secretion protein | -5.70925 |
| RC1_1011 | yes | tatA | Sec-independent protein translocase TatA | 2.81363 |

**COG V: Defense Mechanisms**

| | | | | |
|---|---|---|---|---|
| RC1_2728 | yes | cvaB | colicin V secretion | -3.53106 |
| RC1_1058 | - | norM | multidrug resistance protein NorM, putative | -1.66502 |

**COG not assigned**

| | | | | |
|---|---|---|---|---|
| RC1_0387 | yes | | lipoprotein | -7.32811 |
| RC1_3561 | - | | hypothetical protein | -7.09884 |
| RC1_0388 | yes | | hypothetical protein | -5.8482 |
| RC1_2724 | yes | | hypothetical protein | -5.18816 |
| RC1_2725 | yes | | hypothetical protein | -5.01777 |
| RC1_1712 | yes | | hypothetical protein | -5.00345 |
| RC1_2729 | yes | | hypothetical protein | -4.67853 |
| RC1_1542 | yes | | hypothetical protein | -4.46385 |
| RC1_0033 | - | | hypothetical protein | -4.3855 |
| RC1_3330 | - | | hypothetical protein | -4.2457 |
| RC1_0873 | yes | | hypothetical protein | -4.19825 |
| RC1_2733 | yes | | transposase, is4 family | -3.95379 |
| RC1_3560 | - | | tetrapyrrole | -3.91976 |
| RC1_4075 | - | | ABC transporter substrate-binding protein | -3.7902 |
| RC1_4105 | - | | hypothetical protein | -3.56267 |
| RC1_2354 | - | | hypothetical protein | -3.51915 |
| RC1_4106 | yes | | hypothetical protein | -3.4052 |
| RC1_0034 | - | | hypothetical protein | -3.35299 |
| RC1_3895 | yes | | hypothetical protein | -3.15465 |
| RC1_4126 | yes | | pseudogene | -2.89232 |
| RC1_3192 | yes | | hypothetical protein | -2.79248 |
| RC1_0738 | yes | | PRC1_-barrel domain-containing protein | -2.70468 |
| RC1_1729 | yes | | hypothetical protein | -2.62466 |
| RC1_2949 | - | | hypothetical protein | -2.49189 |
| RC1_0229 | - | | hypothetical protein | -2.30572 |
| RC1_0851 | - | | hypothetical protein | -2.29494 |
| RC1_2245 | - | | hypothetical protein | -2.25132 |
| RC1_1730 | yes | | transposase and inactivated derivative | -2.1943 |
| RC1_2412 | - | | hypothetical protein | -2.1517 |
| RC1_1924 | - | | hypothetical protein | -2.02167 |
| RC1_1425 | - | | hypothetical protein | -1.92138 |
| RC1_1785 | - | | methyl-accepting chemotaxis protein | -1.80057 |

| | | | | |
|---|---|---|---|---|
| RC1_3569 | - | | hypothetical protein | -1.75838 |
| RC1_1766 | - | | hypothetical protein | -1.52148 |
| RC1_1857 | - | | hypothetical protein | -1.47377 |
| RC1_1603 | - | | hypothetical protein | -1.40923 |
| RC1_3212 | - | | hypothetical protein | -1.33566 |
| RC1_3213 | - | | hypothetical protein | -1.33566 |
| RC1_3034 | yes | | hypothetical protein | 1.48073 |
| RC1_3906 | yes | | hypothetical protein | 1.48551 |
| RC1_2345 | yes | | nuclease-like protein | 1.79904 |
| RC1_3863 | - | aer | aerotaxis sensor receptor Aer | 1.81713 |
| RC1_2144 | - | | hypothetical protein | 2.05025 |
| RC1_1520 | - | | hypothetical protein | 2.40306 |
| RC1_3190 | - | | hypothetical protein | 2.47525 |
| RC1_3904 | yes | | hypothetical protein | 2.59141 |
| RC1_1512 | - | | DNA-binding protein | 2.60741 |

**[1]Genes that were previously observed to be differentially expressed during cyst development [25].**
