# Supplementary material for: Mapping the CgrA regulon of Rhodospirillum centenum reveals a hierarchal network controlling Gram-negative cyst development
Source: BMC Genomics. 2015 Dec 16;16:1066. doi: 10.1186/s12864-015-2248-z (PMC4681086; doi:10.1186/s12864-015-2248-z)
Supplement: Additional file 2: — Table S2. A table of all called CgrA ChIP-seq peaks and their locations on the genome. (PDF 286 kb) [file 12864_2015_2248_MOESM2_ESM.pdf]

Table S2: Called CgrA ChIP-Seq peaks

Peak number	Summit position[1]	tags[2]	fold enrichment[3]	downstream gene locus[4]	Gene function/discription	Binding sequence	
1	13181	190	4.44	RC1 0024	ABC transporter ATP-binding protein		
2	23505	354	9.98	RC1 0030 and RC1 0031	hypothetical, tRNA-Ser		
3	45679	1120	15.53	RC1 0056	hypothetical protein	CGTGCCGGTCGGCACC	
4	65606	325	9.43	RC1 0083, RC1 0082	putative prevent-host-death; transcriptional regulator	TGTGCCATCGCTCACC	
5	75343	229	3.33	RC1 0093	phage protein Gp37		
6	124964	210	4.66	RC1 0135	hfq		
7	127435	388	3.39	RC1 0137	formyltetrahydrofolate deformylase		
8	136370	255	4.95				
9	144157	362	7.14	RC1 0155	inosine-5'-monophosphate dehydrogenase		
10	156164	729	6.88	RC1 0163	hypothetical protein		
11	246411	247	6.8	RC1 0255	hypothetical protein		
12	251280	302	7.24	RC1 0261	RNA polymerase sigma-70 factor		
13	271237	214	3.05	RC1 0275	Zn-dependent aminopeptidase		
14	287786	157	5.3	RC1 0287	cytochrome d ubiquinol oxidase	TGTAAGAATTAGAACA	
15	322947	257	7.48	RC1 0310	succinyl-CoA transferase		
16	332539	448	3.19	RC1 0319	nrdD; anaerobic ribonucleoside triphosphate reductase	GGTGCCGGTCGGCACC	
17	343033	118	8.07	RC1 0329	hypothetical protein		
18	376301	842	3.63	RC1 0359	hypothetical protein		
19	391707	5622	20.28	RC1 0373 and RC1 0374	5S ribosomal RNA; 16S ribosomal RNA		
20	427438	996	6.4	RC1 0402	TonB dependent receptor		
21	450698	266	3.51				
22	469137	732	10.47	RC1 0438 and RC1 0439	OxyR regulator; ahpC; alkyl hydroperoxide reductase C	GTGGCGCCAGCACCAC	
23	476604	745	11.74	RC1 0448	rplU; 50S ribosomal protein L21	GTGATCGGTCAGGCAC	
24	521335	748	5.93	RC1 0494	rrf2 family transcriptional regulator		
25	525581	151	7.99	RC1 0498	hypothetical protein		
26	550415	229	5.47	RC1 0518	wsp; surface protein		
27	557106	101	6.08	RC1 0521 and RC1 0522	hypothetical proteins		
28	569533	113	4.07	RC1 0533	hypothetical protein		
29	572214	161	5.99	RC1 0535 and RC1 0534	acyl-CoA dehydrogenase and hypothetical protein		
30	577597	177	3.4	RC1 0538	glycosyl transferase family protein		
31	581825	115	3.28	RC1 0543	polyhydroxyalkanoate synthesis repressor PhaR		
32	583394	141	5.12	RC1 0546	HicA-like protein		
33	598698	581	13.6	RC1 0600	hypothetical protein		
34	611578	126	9.9	RC1 0574	Carbon starvation protein CstA		
35	616185	314	5.43	RC1 0579	hypothetical protein		
36	620871	346	3.7	RC1 0582	DnaJ family, molecular chaperone		
37	623540	153	4.66	RC1 0586	hypothetical protein		
38	642726	622	16.89	RC1 0605	UDP-3-O-[3-hydroxymyristoyl] N-acetylglucosamine deacetylase	GTGCGGTCATCCTCAC	GTGTGACGGCGCAC
39	662797	357	2.9	RC1 0622	mraZ; cell division protein MraZ		

40	667768	284	10.81	RC1 0627	acetyltransferase, GNAT family	
41	675034	120	3.45	RC1 0632 and RC1 0633	hypothetical protein; Sensor histidine kinase	
42	679263	968	8.6			
43	693303	141	4.64	RC1 0643	glyoxalase family protein	
44	718114	98	3.74	RC1 0667	hypothetical protein	
45	730369	377	10.76	RC1 0679->RC1 0683	hypothetical protein; tonB; exbB; exbD; exbD1	
46	739382	4653	11.3	RC1 0695	secE; preprotein translocase subunit SecE	
47	757379	208	6.07	RC1 0706	30S ribosomal protein S12	
48	773930	442	6.61	RC1 0733	rpsM; 30S ribosomal protein S13	
49	783209	328	6.22	RC1 0743	VacJ like lipoprotein	
50	788943	230	4.07	RC1 0747	hypothetical protein	
51	794168	389	5.91	RC1 0754	phosphoesterase	
52	805618	96	6.81	RC1 0767	hypothetical protein	
53	822465	387	10.74	RC1 0783	hypothetical protein	CGTGCGTTTACGCACC
54	825277	192	8.98	RC1 0785 and RC1 0786	molybdopterin biosynthesis protein MoeA	
55	842215	327	4.06	RC1 0799	TonB system biopolymer transport	
56	847580	313	10.71	RC1 0805	glutamine amidotransferase, class-II	
57	872209	2932	12.21			
58	880371	90	4.81	RC1 0834	hypothetical protein	
59	883056	123	7.36	RC1 0836	rho; pseudogene	
60	888348	428	6.86	RC1 0844	hypothetical protein	GTGTGACAGGGGTCAC
61	891848	216	12.39	RC1 0848 and RC1 0849	transcriptional regulator, LuxR family proteins	
62	896784	869	6.25	RC1 0855	hypothetical protein	
63	904735	401	3.61	RC1 0864	glgP; glycogen phosphorylase	
64	933930	303	4.13	RC1 0890	flgB; flagellar basal-body rod protein	
65	944039	355	6.45	RC1 0898	methyl-accepting chemotaxis protein	
66	956413	653	10.69	RC1 0907 and RC1 0908	GcrA cell cycle regulator; sensory box sensor histidine kinase	
67	965100	360	6.37	RC1 0918	hypothetical protein	
68	970768	260	3.22	RC1 0921	nrdJ; ribonucleotide-diphosphate reductase	
69	982607	181	7.45	RC1 0932	peptidase M48	
70	988150	197	5.49	RC1 0938	tRNA (5-methylaminomethyl-2-thiouridylate)-methyltransferase	CGTGGTCGGCATCACG
71	989450	544	11.78	RC1 0942	hemolysin activation	
72	1016893	667	6.19	RC1 0955	cheY; chemotaxis protein CheY	
73	1082515	135	4.63	RC1 1017	hypothetical protein	
74	1108774	90	3.98	RC1 1044	fusion protein of y4aC and y4aD	
75	1116224	152	3.84	RC1 1052	hypothetical protein	
76	1141270	614	7.29	RC1 1075	carbon monoxide dehydrogenase	GGTGAAGAGCTGCACC
77	1149011	397	4.65	RC1 1086	putative helix-turn-helix XRE-like protein	
78	1178336	143	4.25	RC1 1132	phage baseplate protein	
79	1197963	1141	10.04	RC1 1155	tRNA-Asn	GGGATGTTCACGGCAC
80	1205610	141	3.56	RC1 1163->RC1 1165	ilvD; dihydroxy-acid dehydratase; hypothetical; mltB	
81	1267295	134	3.71	RC1 1210	dnaE; DNA polymerase III subunit alpha	
82	1275853	259	5.49			
83	1300913	1091	4.94	RC1 1247	clpX; ATP-dependent protease ATP-binding subunit	
84	1309586	412	12.7	RC1 1255	hypothetical protein	

85	1312790	91	6.08	RC1_1261	frnE; DSBA-like thioredoxin family protein	
86	1322096	220	7.2	RC1_1268	bcsA; chalcone synthase	GGTGACACCCCGCACG
87	1328826	175	5.99	RC1_1275	fixH; nitrogen fixation protein fixH	GGGCCGCCGCGATCAC
88	1351173	477	16	tRNA		
89	1369638	647	9.9	tRNA		
90	1371379	157	3.58	tRNA		
91	1373357	1421	8.78			
92	1387934	664	5.55	RC1_1346	expG; exopolysaccharide II synthesis transcriptional activator ExpG	
93	1398967	356	13.13	RC1_1357	ornithine decarboxylase	
94	1432677	313	4.43	RC1_1393	flgI; flagellar P-ring protein	
95	1446907	629	5.3	RC1_1407	narL; nitrate/nitrite response regulator protein NarL	
96	1451808	304	10.73	RC1_1412	otsB; trehalose-phosphatase	
97	1469755	206	3.75	RC1_1428 and RC1_1429	transcriptional regulator, PadR family; HlyD family secretion protein	
98	1477882	754	3.71	RC1_1435	hypothetical protein	
99	1499217	259	7.51	RC1_1456	aguA; agmatine deiminase	
100	1517456	741	8.17	RC1_1474	capD; polysaccharide biosynthesis protein	
101	1529641	175	2.88	RC1_1484	phage-related hypothetical protein	
102	1545022	682	4.9	RC1_1499	guanosine polyphosphate phosphohydrolase	
103	1557880	84	4.32	RC1_1514	hypothetical protein	
104	1576530	424	9.42	RC1_1531	rpmG; 50S ribosomal protein L33	
105	1581442	423	3.37	RC1_1535 and RC1_1536	hypothetical protein; transcriptional regulator, MarR family	
106	1633372	200	5.4	RC1_1588	alcohol dehydrogenase (acceptor)	CGTGACCGCCGTCACA
107	1643603	301	15.14		tRNA-val	
108	1656266	141	7.34	RC1_1608	mrcA; penicillin-binding protein 1A	
109	1658168	390	7.33	RC1_1609	N-acetylmuramoyl-L-alanine amidase	
110	1667388	276	6.54	RC1_1620	hypothetical protein	
111	1688558	172	7.48	RC1_1636	hypothetical protein	
112	1724403	98	3.28	RC1_1674	hypothetical protein	AGTGACGCACATCACA
113	1725713	270	6.74		tRNA-Pro	
114	1741925	1210	7.95	RC1_1693	phasin family protein	
115	1744403	250	4.85	RC1_1695	dacA; D-alanyl-D-alanine carboxypeptidase	
116	1754247	173	5.99	RC1_1703	agmR; glycerol metabolism activator	
117	1764369	264	8.3	RC1_1717	ornithine decarboxylase	
118	1774703	243	3.88	RC1_1724	RNA polymerase sigma-32 factor	
119	1795752	239	6.69	RC1_1745 and RC1_1746	inner membrane protein YbaN; transcriptional regulator hexR	
120	1808567	176	3.01			
121	1839119	206	6.65	RC1_1783	hypothetical protein	
122	1844291	393	8.47	RC1_1788	phosphoserine aminotransferase	
123	1846272	634	18.23	RC1_1790	hypothetical protein	
124	1853367	371	4.4	RC1_1796	ftsH; ATP-dependent metalloprotease	
125	1856222	406	5.61	RC1_1799	outer membrane lipoprotein omp16	
126	1860467	105	4.24			
127	1865631	81	4.45	RC1_1810	pseudogene	
128	1895932	322	9.51			
129	1913334	316	3.57			

130	1933045	465	11.76				
131	1951141	1570	12.13	RC1_1890	pleC; non-motile and phage-resistance protein		
132	1954443	244	7.17				
133	1956947	274	6.2	RC1_1898	proC; pyrroline-5-carboxylate reductase		
134	1980148	475	22.33	RC1_1927	CRISPR-associated protein		
135	1985122	392	5.94				
136	2018430	237	4.86		tRNA-Lys		
137	2026111	903	6.09	RC1_1970	hypothetical protein		
138	2033883	2028	11.15	RC1_1975	acetolactate synthase large subunit		
139	2035345	106	5.95	RC1_1978	hypothetical protein		
140	2043208	489	17.93	RC1_1985	hypothetical protein		
141	2053481	203	5.69	RC1_1993->RC1_2001	R body gene cluster; RNA polymerase sigma-70 factor		
142	2056165	570	9.13	tmRNA		GTGCCAACGACAACAC	
143	2059698	380	5.08	RC1_2006	RNA polymerase sigma factor RpoD	TGTGTTCCCGGGAACA	GGTGCCAACGACAACAC
144	2065667	242	6.54	RC1_2014	carB; carbamoyl-phosphate synthase		
145	2068963	433	8.82	RC1_2015	transcription elongation factor GreA		
146	2079714	80	5.36	RC1_2026	deoxyribodipyrimidine photolyase family protein		
147	2097388	104	4.52	RC1_2037	hypothetical protein		
148	2099544	294	4.48	RC1_2040	pseudogene		
149	2107060	203	5.63	RC1_2047	cstS2; protein CstS2		
150	2128671	218	5.27	RC1_2070	30S ribosomal protein S21		
151	2135320	415	4.97	RC1_2078	bchG; bacteriochlorophyll/chlorophyll a synthase		
152	2150061	375	10.8	RC1_2091	crtE geranyltransferase	GTGGGCTCCAGGTCAC	
153	2152479	3184	10.24	RC1_2093	bchC; 2-desacetyl-2-hydroxyethyl bacteriochlorophyllide A		
154	2176319	110	2.49				
155	2178804	2669	14.35	RC1_2117	aerR; coenzyme B12-binding aerobic repressor		
156	2206791	561	6.07	RC1_2140	hypothetical protein	GTGGAAAAGCCTCCAC	
157	2225603	346	4.71	RC1_2161	cspG; Cold shock-like protein		
158	2228526	106	3.49	RC1_2163	hypothetical protein		
159	2235640	552	9.98	RC1_2169	RNA polymerase sigma-32 factor		
160	2241884	573	10.43	RC1_2176	fpr; ferredoxin--NADP reductase		
161	2271226	724	11.38	RC1_2202	pentapeptide repeat-containing protein	CGTGGGGGAGCGCACC	
162	2291842	763	5.76	RC1_2223	CarD-like transcriptional regulator protein	GTGAGGGCCCGCGCAC	
163	2293859	896	14.75	RC1_2225	RpoH RNA polymerase sigma-32	GGTGCGCAGCTACACC	GGTGCGCTTCTCCACC
164	2296624	146	4.06				
165	2304852	70	3.97	RC1_2235	sodium/hydrogen exchanger family protein		
166	2323083	83	4.54	RC1_2253	transcriptional regulatory protein TyrR		
167	2355710	387	7.3	RC1_2280	merA1; mercuric reductase		
168	2399154	326	6.68	RC1_2326	hypothetical protein		
169	2402510	183	4.12	RC1_2328	TRAP transporter solute receptor		
170	2450642	209	8.53	tRNA			
171	2464553	314	3.52				
172	2504603	249	6.2	RC1_2427	hypothetical protein		
173	2508242	199	3.82	RC1_2430	phaZ; intracellular PHB depolymerase		
174	2521037	331	7.83	RC1_2443	sodium/hydrogen exchanger family protein		

175	2530875	341	4.52	RC1_2448	feoB; ferrous iron transport protein B		
176	2544297	165	3.67	RC1_2466	integral membrane protein		
177	2552947	271	5.04				
178	2574586	620	18.86	tRNA			
179	2575993	163	4.6	RC1_2499	lonD; ATP-dependent protease		
180	2588008	281	3.72	RC1_2513 and RC1_2514	psp_ transcription activator; hypothetical protein		
181	2596748	268	9.02	RC1_2527	hypothetical protein		
182	2605488	132	5.38	RC1_2533	succinoglycan biosynthesis transport protein ExoP		
183	2612183	103	3.94	RC1_2541	sensor histidine_ kinase RegB		
184	2613699	382	11.21	RC1_2542	regA; photosynthetic apparatus regulatory protein	GTGCCGACACAGCCAC	
185	2633804	1076	11.8	RC1_2564	RND family efflux transporter MFP subunit		
186	2639274	1119	18.53	RC1_2567	hypothetical protein		
187	2657803	379	10.25	RC1_2586	glycerol metabolism activator AgmR	GGTGCGCAGCCGCACC	
188	2693709	147	3.22	RC1_2619	hypothetical protein		
189	2704890	934	17.1	rRNA			
190	2732706	447	5.29	RC1_2654	yicH; inner membrane protein		
191	2746555	231	4.39	RC1_2668	hypothetical protein		
192	2761030	1281	7.26	RC1_2686	isocitrate lyase		
193	2769588	353	2.59	RC1_2696	long-chain acyl-CoA synthetase		
194	2783067	89	5.29	RC1_2702	gdhA; NAD-glutamate dehydrogenase	TGTAATGGTGGTCACA	
195	2811355	388	7.08	RC1_2730	hypothetical protein		
196	2813591	996	17.11			TGTCTATTCCTAAACA	TGTTGGGCGCTTGACA
197	2833909	82	4.87	RC1_2754	hypothetical protein		
198	2843825	251	6.13	RC1_2761	hypothetical protein		
199	2860303	323	8.97				
200	2866777	208	5.92	RC1_2784	succinyldiaminopimelate aminotransferase		
201	2902912	441	3.65	RC1_2820	translation initiation factor IF-3		
202	2944330	70	4.18	RC1_2860	hypothetical protein		
203	2947649	201	6.57	RC1_2865	pfpL; peptidase C56		
204	2953886	273	12.75	tRNA			
205	2964863	188	6.73				
206	2979293	168	3.96	RC1_2901	hypothetical protein		
207	2986818	459	20.32	RC1_2909	hypothetical protein		
208	3002239	371	7.65	RC1_2923	outer membrane protein		
209	3026783	725	11.01	RC1_2951	transcriptional regulator, LysR family protein		
210	3028765	169	7.67	RC1_2952	drug resistance transporter Bcr		
211	3033947	1603	15.58				
212	3055127	303	3.12	RC1_2975	hemolysin containing CBS domain		
213	3058937	326	14.95	RC1_2980 and RC1_2981	RNA polymerase sigma-70 factor; transcriptional activator ChrR		
214	3062633	350	4.56	RC1_2985	hypothetical protein		
215	3077035	152	5.5	RC1_3002	hypothetical protein		
216	3082995	392	3.46	RC1_3006	pleC; non-motile and phage-resistance protein		
217	3090094	343	9.8	RC1_3011	cytochrome c biogenesis protein CcmA		
218	3092050	188	7.82	RC1_3013	peptidase M23		
219	3104158	337	6.53	RC1_3027	response regulator receiver domain-containing protein		

220	3122811	218	4.58	RC1 3050	molybdopterin biosynthesis protein MoeB	
221	3126854	660	9.01	RC1 3055	30S ribosomal protein S20	
222	3137383	233	8.2	RC1 3065	gyrB; DNA gyrase subunit B	
223	3160514	255	13.6	RC1 3084	transposase, is4 family	
224	3180241	672	17.29	RC1 3102	thiO glycine oxidase	AGTGTATGATGCCACC
225	3197337	427	6.42	RC1 3120	hypothetical protein	
226	3205221	114	5.06	RC1 3128	hypothetical protein	
227	3209097	123	5.43	RC1 3132	hypothetical protein	
228	3226466	152	5.96	RC1 3143	Alkaline Protease, putative	
229	3231218	191	8.05	RC1 3145	hypothetical protein	
230	3251870	278	4.64			
231	3256474	112	4.63	RC1 3168	diguanylate cyclase/phosphodiesterase	
232	3285358	174	6.32			
233	3292206	1307	11.65	RC1 3195	glutamate synthase	
234	3300222	500	6.13	RC1 3204	yfiA; ribosomal subunit interface protein	
235	3302772	152	4.26	RC1 3208	ABC transporter substrate-binding protein	
236	3304643	166	4.18	RC1 3210	hypothetical protein	
237	3318646	461	20.92	RC1 3323	hypothetical protein	
238	3359542	293	3.16	RC1 3260	tme; malic enzyme	
239	3373866	385	13.26	RC1 3272	hypothetical protein	
240	3414595	232	6.18	RC1 3313	hypothetical protein	
241	3416853	220	5.02	RC1 3317	hypothetical protein	
242	3428740	351	3.4	RC1 3332	TonB-dependent receptor	
243	3441334	346	4.77			
244	3446199	916	18.59	RC1 3342	complex hybrid signal transduction protein	TGTAGTTCGAGTAACA
245	3459566	159	8.05	RC1 3349	sensor histidine kinase	GTGCCCAGCAGGGCAC
246	3468519	241	4.52	RC1 3357 and RC1 3358	hypothetical protein; diguanylate cyclase/phosphodiesterase	
247	3471555	188	6.26	RC1 3359	hypothetical protein	
248	3485410	141	4.03	RC1 3373	entericidin EcnAB family protein	
249	3492954	390	29.06	RC1 3384	integral membrane protein	
250	3504607	94	5.45	RC1 3394	hypothetical protein	
251	3537974	178	4.1			
252	3560530	167	4.38	RC1 3450	hypothetical protein	
253	3571265	251	11.15	RC1 3460	N-acetylmuramoyl-L-alanine amidase	
254	3579765	205	7.41	RC1 3468	hypothetical protein	
255	3589009	1336	12.7	RC1 3478	hypothetical protein	
256	3596271	719	7.26	RC1 3482	CRISPR-associated protein	
257	3602455	636	11.95			
258	3607786	556	4.29			
259	3620366	143	5.93	RC1 3503	septum site-determining protein MinC	
260	3630392	558	13.76	RC1 3515	otsA; alpha,alpha-trehalose-phosphate synthase	
261	3632259	345	8.28	tRNA		
262	3655026	202	4.82	RC1 3537	translation elongation factor G	
263	3658067	2035	8.44	rRna		
264	3668466	364	3.59	RC1 3547	hemH; ferrochelatase	

265	3695744	625	3.73	RC1 3568	transcriptional regulator MhpR		
266	3702807	261	6.9	RC1 3577	pseudogene		
267	3727540	92	4.11	RC1 3600	hypothetical protein		
268	3750131	396	5.25				
269	3767538	77	4	RC1 3639	hypothetical protein		
270	3774471	438	3.8				
271	3821715	123	5.45	RC1 3691	nifA; Mo/Fe nitrogenase specific transcriptional regulator		
272	3835144	5548	20.38	rRNA			
273	3902577	622	11.5	RC1 3759	flaG; flagellin protein FlaG		
274	3907316	1571	7.04	RC1 3763	flagellar protein Laf1	GTGACCTCAAGACCAC	
275	3931549	202	9.3	RC1 3783	hypothetical protein		
276	3935196	1287	11.32	RC1 3785	hypothetical protein	GTGTGAAGCAGTTCAC	GTGAAGCAGTTCACAC
277	3945671	499	7.1	RC1 3796	hypothetical protein		
278	3994749	353	12.52	RC1 3822	putative Ig domain-containing protein		
279	4008520	868	8.45	RC1 3833	exbB; biopolymer transport ExbB protei		
280	4041017	284	6.86	RC1 3864	sbp; sulfate-binding protein;		
281	4045365	156	10.08	RC1 3868	hypothetical protein		
282	4058859	273	9.36	RC1 3881	diguanylate cyclase	GTGTCTTTACCGGCAC	GTGGTCTCACAGGGCAC
						GTGGGAGGTGCCGCAC	
283	4088467	161	3.99	RC1 3900	transcriptional regulator, TetR family		
284	4091715	881	6.79	RC1 3905	pseudogene		
285	4123825	721	14.26	RC1 3923	diguanylate cyclase		
286	4141975	238	4.76	RC1 3937	acetyltransferase		
287	4157557	344	3.57	RC1 3954	phosphate-selective porin O and P		
288	4164077	445	4.8	RC1 3961	3-isopropylmalate dehydratase small subunit		
289	4213424	256	3.92	RC1 4002	colanic acid biosynthesis glycosyl transferase WcaI		
290	4233550	199	4.63	RC1 4020	hypothetical protein		
291	4254560	283	3.56	RC1 4036	cytochrome D ubiquinol oxidase, subunit I		
292	4259532	89	5.99	RC1 4041	TonB-dependent receptor		
293	4267341	144	6.48	RC1 4049	RND efflux system, outer membrane lipoprotein, NodT		
294	4332241	260	5.05	RC1 4105	hypothetical protein		
295	4340867	132	4.44	RC1 4117	HNH endonuclease family protein		

[1]Genomic location of each CgrA ChIP-seq peak. Summit: location within each peak with the highest read count. (*R. centenum* genome NCBI Reference Sequence: NC 011420.2,

[2]Number of tags (reads) in peak region.

[3]Fold enrichment for peak region against random Poisson distribution with local lambda.

[4]Gene downstream of each CgrA ChIP-seq peak.

highlighted genes: genes that were previously observed to be differentially expressed during cyst development [25].
